# Mesenchymal stromal cells equipped by IFNα empower T cells with potent anti-tumor immunity

**DOI:** 10.1038/s41388-022-02201-4

**Published:** 2022-02-10

**Authors:** Tao Zhang, Yu Wang, Qing Li, Liangyu Lin, Chunliang Xu, Yueqing Xue, Mingyuan Hu, Yufang Shi, Ying Wang

**Affiliations:** 1 CAS Key Laboratory of Tissue Microenvironment and Tumor, Shanghai Institute of Nutrition and Health, University of Chinese Academy of Sciences, Chinese Academy of Sciences, Shanghai, 200031 China; 2 The Third Affiliated Hospital of Soochow University, State Key Laboratory of Radiation Medicine and Protection, Institutes for Translational Medicine, Soochow University Medical College, Suzhou, Jiangsu 215123 China

**Keywords:** Cancer microenvironment, Immunotherapy

## Abstract

Cancer treatments have been revolutionized by the emergence of immune checkpoint blockade therapies. However, only a minority of patients with various tumor types have benefited from such treatments. New strategies focusing on the immune contexture of the tumor tissue microenvironment hold great promises. Here, we created IFNα-overexpressing mesenchymal stromal cells (IFNα-MSCs). Upon direct injection into tumors, we found that these cells are powerful in eliminating several types of tumors. Interestingly, the intra-tumoral injection of IFNα-MSCs could also induce specific anti-tumor effects on distant tumors. These IFNα-MSCs promoted tumor cells to produce CXCL10, which in turn potentiates the infiltration of CD8^+^ T cells in the tumor site. Furthermore, IFNα-MSCs enhanced the expression of granzyme B (GZMB) in CD8^+^ T cells and invigorated their cytotoxicity in a Stat3-dependent manner. Genetic ablation of Stat3 in CD8^+^ T cells impaired the effect of IFNα-MSCs on GZMB expression. Importantly, the combination of IFNα-MSCs and PD-L1 blockade induced an even stronger anti-tumor immunity. Therefore, IFNα-MSCs represent a novel tumor immunotherapy strategy, especially when combined with PD-L1 blockade.

## Introduction

The tumor microenvironment contains multiple types of immune cells, such as T cells, B cells, macrophages, and neutrophils, as well as tumor stromal cells, including fibroblasts, endothelial cells, and mesenchymal stromal cells [1]. These nonmalignant cells not only facilitate tumor growth and progression, but also strongly affect the efficiency of various cancer treatments [2–4]. Strategies targeting immune checkpoints, such as PD-L1 and CTLA-4, have been shown to restore the function of exhausted CD8^+^ T cells and have demonstrated impressive efficacy in some patients suffering from various cancer types. However, the majority of cancer patients do not acquire durable benefit [5]. Therefore, novel tumor microenvironment modulating strategies are still to be formulated to effectively eradicate tumors.

It has been demonstrated that preexisting infiltrated T cells in the tumor microenvironment are a good prognosis marker for cancer treatment [6–8]. According to the immune contexture, tumors can be classified into “hot” (inflamed) and “cold” (non-inflamed) tumors [9, 10]. The immune features of “hot” tumors are a good indicator for the utility of immune checkpoint blockades. However, the abundance of stromal cells within tumors could significantly affect the infiltration of T cells in the tumors [11]. When a tumor was found to possess less infiltrated immune cells, more resident immunosuppressive cells, or abundant immune cells in surrounding tissues, a poor prognosis of the treatment of immune checkpoint blockade is often given [10, 12]. Indeed, patients who have impaired ability to deploy immune cells or are lack of a balance between CD8^+^ T cells and tumor burden responded weakly to immune checkpoint blockade therapies [13]. Thus, reinvigorating immune cells in tumors that are featured by high PD-L1 using type I interferon (IFN) could lead to proper T cell activation, a strategy that even could be employed as a combination with immune checkpoint blockades [14].

By deciphering the interplay among PD-L1, IFNs, and T cell function, studies have demonstrated that type I IFN signaling is indispensable for rejection of tumor cells via the initiation of anti-tumor T cell responses [15, 16]. Moreover, PD-L1 expression is mainly determined by IFNs, subsequently impairing the ability of T cells to eradicate tumor cells. However, the expression of type I IFNs in the tumor microenvironment is limited [14]. Indeed, the efficacy of conventional chemotherapeutics [17], targeted therapies [18], radiotherapy [19, 20], and immunotherapy [21, 22] could rely upon the induction of type I IFN signaling. As such, exogenous IFNα administration has been attempted to treat various tumor types, however, its short half-life and collateral toxicity restrict its clinical applications [23, 24]. Therefore, remodeling tumor microenvironment by reinforcing sustained type I IFN signals may be a feasible way to enhance immune checkpoint therapy responsiveness.

In this study, we used several tumor models to define the role of IFNα-MSCs in defending spontaneous tumors and eradicating established tumors in mouse models. We found that IFNα-MSC administration greatly inhibited tumor progression through enhancing the recruitment of CD8^+^ T cells and their cytotoxicity. Interestingly, such suppression is far-ranging and tumor specific. We found that IFNα-MSCs induced enriched CXCL10 expression in tumor cells which is responsible for the chemotaxis of CD8^+^ T cells to the tumor site. IFNα derived from IFNα-MSCs increased the expression of GZMB in CD8^+^ T cells through Stat3 signaling. Such concerted actions induced by IFNα-MSCs reinvigorated anti-tumor response of CD8^+^ T cells. More importantly, IFNα-MSCs, in combination with α-PD-L1 optimize the activation of CD8^+^ T cells to control tumor. These findings may have important implications for developing more effective anti-tumor immunotherapies.

## Results

### Low level of type I IFN signaling is associated with poor prognosis of melanoma

**Fig. 1:**
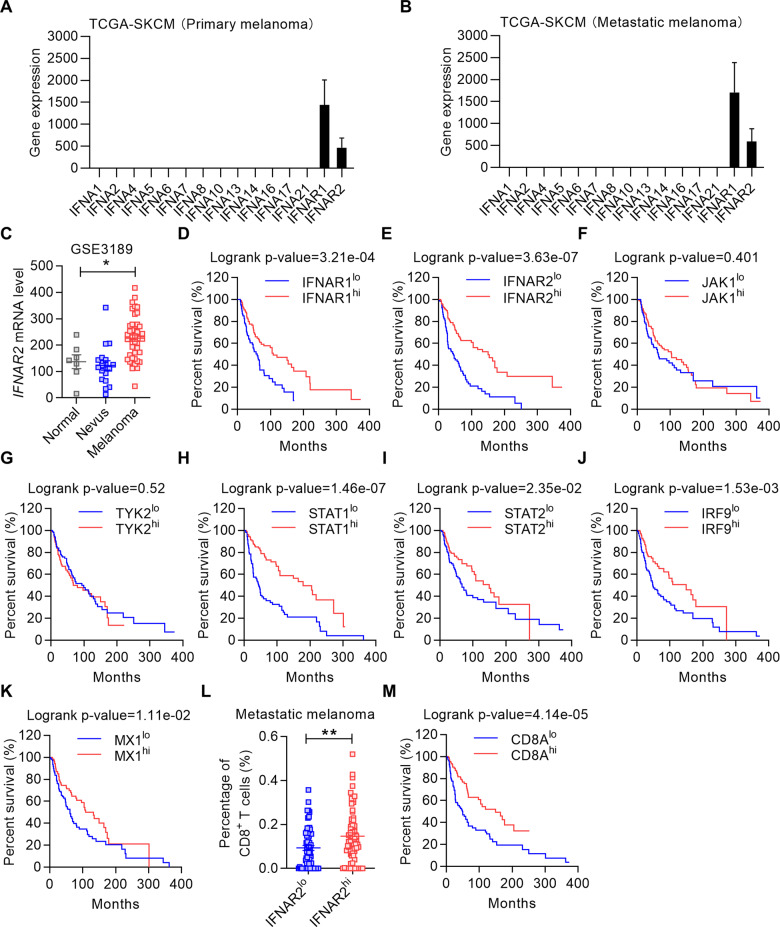
Lack of type I IFN signaling is a negative prognostic indicator of melanoma patients. **A**, **B** The expressions of all *IFNA* subtypes and *IFNAR* in human primary melanoma (**A**) and metastatic melanoma (**B**). **C** Comparison of *IFNAR2* expression in normal skin, nevus, and melanoma tissues using the GSE3189 dataset. **D**–**K** The overall survival curves of patients with melanoma. Patients were stratified (cutoff at 25%) according to the expression of *IFNAR1* (**D**), *IFNAR2* (**E**), *JAK1* (**F**), *TYK2* (**G**), *STAT1* (**H**), *STAT2* (**I**), *IRF9* (**J**) and *MX1* (**K**) in the TCGA-SKCM dataset. **L** The percentages of CD8^+^ T cells in patients with metastatic melanoma classified according to the expression of *IFNAR2*. Patients were stratified into *IFNAR2*^*lo*^ and *IFNAR2*^*hi*^ cohorts (cutoff at 25%). The percentages of CD8^+^ T cells in the metastatic melanoma tissues were enumerated using CIBERSORT. **M** The overall survival analysis of melanoma patients with *CD8A*^*lo*^ and *CD8A*^*hi*^ expression (cutoff at 25%). Data are shown as means ± SEM. *p < 0.05 and **p < 0.01.

To investigate the correlation between type I IFN signaling and melanoma development, we used the Cancer Genome Atlas (TCGA) to analyze the expression patterns of *IFNA* and *IFNA* receptor (*IFNAR*) in melanoma patients. Although all the *IFNA* transcripts were barely detectable, high levels of *IFNAR1* and *IFNAR2* expression were observed in both primary and metastatic melanoma (Fig. 1A, B and Supplementary Fig. S1A, B). We further compared the expressions of *IFNAR1* and *IFNAR2* in normal skins, nevus tissues, and melanoma tissues using a published dataset (GSE3189) and found that the expressions of *IFNAR1* and *IFNAR2* are significantly higher in melanoma as compared to normal skin and nevus tissues (Fig. 1C and Supplementary Fig. S1C) [25]. These observations suggest that the alterations of *IFNAR* expression may modulate melanoma progression.

Distinct from janus kinase 1 (*JAK1*) and tyrosine kinase 2 (*TYK2*), the abundance of *IFNAR1*, *IFNAR2*, signal transducer and activator of transcription 1 (*STAT1*), signal transducer and activator of transcription 1 (*STAT2*), interferon regulatory factor 9 (*IRF9*), and MX dynamin-like GTPase 1 (*MX1*) are predictors of good prognosis of melanoma (Fig. 1D–K). Since all these genes were related to type I IFN activities, we employed cell-type identification by estimating relative subsets of RNA transcripts (CIBERSORT) algorithm to link the expression of *IFNAR* to CD8^+^ T cell infiltration [26]. We found that the expression of *IFNAR2*, but not *IFNAR1*, is correlated with the enrichment of infiltrated CD8^+^ T cells in the metastatic melanoma tissue microenvironment (Fig. 1L and Supplementary Fig. S1D). Patients with high expression of *CD8A* in the tumor site exhibited a higher overall survival rate (Fig. 1M). Taken together, these data demonstrate that type I IFN signatures in the tumor microenvironment predict a good prognosis of melanoma.

### IFNα-MSCs elicit anti-tumor activities to several tumor types

**Fig. 2:**
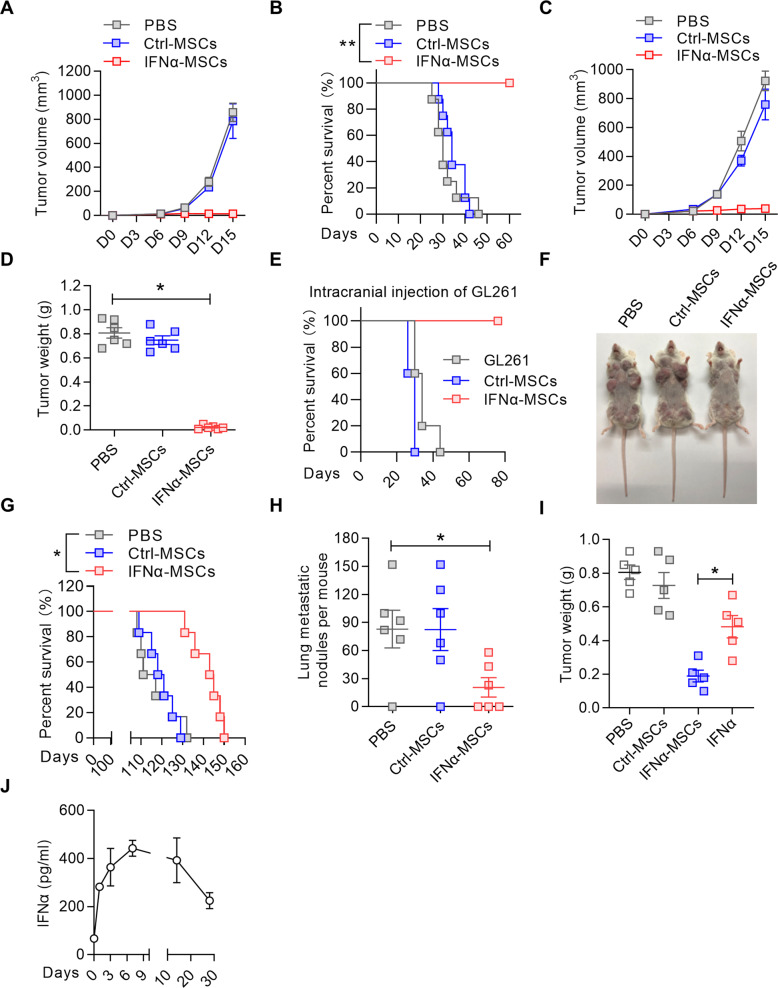
IFNα-MSCs elicit powerful anti-tumor activity. **A** Impact of IFNα-MSCs on B16F0 melanoma progression. B16F0 cells (1.0 × 10^6^) were intramuscularly injected into the right outer thigh of mice. Mice received Ctrl-MSCs (1.0 × 10^6^) or IFNα-MSCs (1.0 × 10^6^) every 3 days starting on day 3. On days 6, 9, 12, and 15, tumor size was measured. **B** The survival curves of B16F0 melanoma bearing mice treated with PBS, MSCs, or IFNα-MSCs (*n* = 8). **C** Impact of IFNα-MSCs on MC38 tumor progression. Mice were intramuscularly injected with MC38 cells (1.0 × 10^6^) and treated with Ctrl-MSCs (1.0 × 10^6^) or IFNα-MSCs (1.0 × 10^6^) every 3 days starting on day 3. On days 9, 12, and 15, tumor size was measured. **D** Mass of MC38 tumors treated with PBS, Ctrl-MSCs, or IFNα-MSCs (*n* = 6). **E** Impact of IFNα-MSCs on the survival of mice bearing GL261 tumors. Mice were intracranially co-injected with GL261 cells (2 × 10^5^) and Ctrl-MSCs or IFNα-MSCs at a ratio of 300:1 (*n* = 5). **F** Visualization of tumor burden in MMTV-PyMT mice treated with PBS, Ctrl-MSCs, or IFNα-MSCs. MMTV-PyMT mice at 4 weeks old received Ctrl-MSCs (3.0 × 10^5^) or IFNα-MSCs (3.0 × 10^5^) twice a week. **G** The impact of IFNα-MSCs on the survival of MMTV-PyMT mice (*n* = 6). Data were pooled from two independent experiments. **H** The number of metastatic tumor nodules in the lungs of MMTV-PyMT mice treated with PBS, Ctrl-MSCs, or IFNα-MSCs (*n* = 6). **I** Comparison of the therapeutic effect of IFNα-MSCs and IFNα on melanoma. Mice were intramuscularly injected with B16F0 cells (1.0 × 10^6^) and received single dose of Ctrl-MSCs (1.0 × 10^6^), IFNα-MSCs (1.0 × 10^6^), or IFNα (5 μg) on day 5. The tumors were weighed on day 14 (*n* = 5). **J** IFNα concentration in the serum of melanoma bearing mice treated with IFNα-MSCs. Mice were co-injected with B16F0 cells (1.0 × 10^5^) and IFNα-MSCs (1.0 × 10^6^). IFNα in the serum was assayed at indicated times by ELISA. Data are presented as means ± SEM. *p < 0.05 and **p < 0.01.

Given a desert signature of type I IFN in the microenvironment of melanoma, we brought IFNα to the tumor site by employing MSCs constitutively expressing IFNα (IFNα-MSCs) to treat mice with tumors. These MSCs continuously release IFNα locally and thus avoid the concentration fluctuations and associated side effects of systemic administration of IFNα [27, 28]. Using ELISA assay, we verified that IFNα-MSCs robustly produced IFNα in vitro (Supplementary Fig. S2A). We investigated the anti-tumor effects of these IFNα-MSCs in several tumor models in vivo. In a mouse melanoma model, B16F0 were intramuscularly injected into the right outer thigh of C57BL/6 mice. IFNα-MSCs or Ctrl-MSCs were locally injected into peritumoral tissue every 3 days starting on day 3 (Fig. 2A). We found that IFNα-MSCs completely inhibited melanoma growth. In comparison with PBS and Ctrl-MSC treatment, administration of IFNα-MSCs blocked tumor growth and dramatically prolonged the survival of mice bearing melanoma (Fig. 2B). We further utilized IFNα-MSCs to treat colon carcinoma established by the MC38 cell line and found that IFNα-MSCs also exerted a dramatic suppression on tumor growth as measured by tumor volume (Fig. 2C) and tumor weight (Fig. 2D). In a mouse glioma model, GL261 cells were intracranially injected together with Ctrl-MSCs or IFNα-MSCs at a ratio of 300:1. At the time point when all PBS and Ctrl-MSC treated mice reached the point to be euthanized, all mice received IFNα-MSC treatment remain alive (Fig. 2E). These results clearly demonstrate that IFNα-MSCs could exert powerful anti-tumor effect on tumor growth.

We further tested the therapeutic effect of IFNα-MSCs in the B16F10 melanoma model. B16F10 cells were *i.v.* injected into C57BL/6 mice. On day 7, these mice received a single dose of IFNα-MSC infusion. We found that IFNα-MSC treatment significantly inhibited B16F10 melanoma colonization in the lung (Supplementary Fig. S2B–D) and prolonged the survival of tumor bearing mice (Supplementary Fig. S2E). To extend our findings to spontaneously developed tumors, we used IFNα-MSCs to treat MMTV-PyMT mice, which develop spontaneous mammary tumors. At 4 weeks old, the MMTV-PyMT mice were *i.v*. administered with PBS, Ctrl-MSCs, or IFNα-MSCs twice a week. IFNα-MSC administration dramatically decreased the tumor burden of MMTV-PyMT mice and extended their survival (Fig. 2F, G), suggesting a robust anti-tumor effect of IFNα-MSCs. This was consolidated by the observation that IFNα-MSC administration also resulted in a dramatic reduction of tumor metastasis to the lungs (Fig. 2H).

As IFNα has been approved for treating several neoplasms, we compared the anti-tumor effects of IFNα and IFNα-MSCs, and found that IFNα-MSCs exhibited a much more powerful suppression on tumor growth than that of intra-tumoral injection of IFNα (Fig. 2I). By monitoring the serum level of IFNα in IFNα-MSC-treated mice, we found that administration of IFNα-MSCs could sustainably elevate IFNα level in the serum, even on day 28 (Fig. 2J). Distinct from the flu-like symptoms induced by IFNα, IFNα-MSC treatment has no influence on mouse body weight, temperature, and leukocyte number in blood (Supplementary Fig. S2F–H). Therefore, IFNα-MSCs exert extensive anti-tumor effects with no noticeable toxicity.

### IFNα-MSCs impose tumor specific abscopal anti-tumor effect

To investigate the mechanism(s) underlying the tumoricidal effect of IFNα-MSCs, we firstly used their conditioned medium to treat B16F0 cells. Although the conditioned medium of IFNα-MSCs slightly suppressed the tumor growth in vitro, such inhibitory effect may not be the major reason to suppress tumor in vivo (Supplementary Fig. S3A). Consistently, we found that IFNα, even at very high dose, has very limited effects on tumor growth (Supplementary Fig. S3B). Furthermore, no significant influence on cell cycle and apoptosis was observed in B16F0 cells treated with IFNα or conditioned medium of IFNα-MSCs (Supplementary Fig. S3C, D). Thus, the anti-tumor activities of IFNα-MSCs are independent of the direct killing effect of IFNα on tumor cells.

**Fig. 3:**
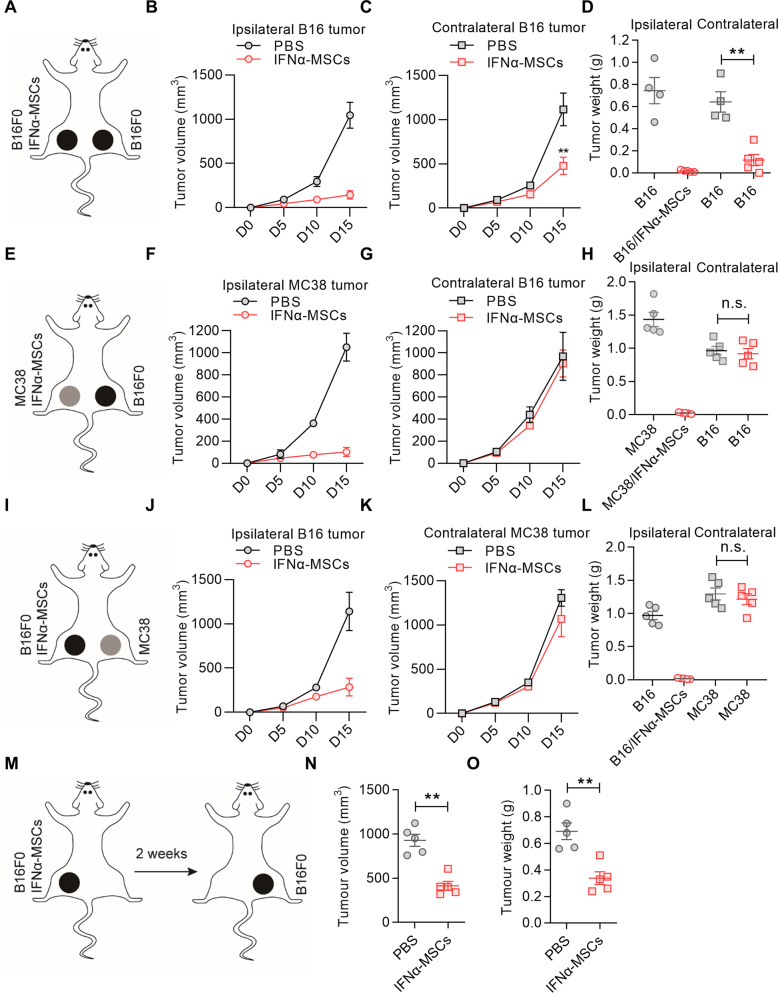
IFNα-MSCs impose systemic specific anti-tumor effect. **A** Schematic representation of the impact of IFNα-MSCs on abscopal tumors. Mice received the co-injection of B16F0 cells (1.0 × 10^6^) and IFNα-MSCs (1.0 × 10^6^) in the left outer thigh and simultaneously inoculated with B16F0 cells (1.0 × 10^6^) in the right outer thigh. **B**, **C** Tumor volumes of ipsilateral (**B**) and contralateral (**C**) B16 tumors treated with or without IFNα-MSCs. **D** Tumor weight of ipsilateral and contralateral B16 tumors treated with or without IFNα-MSCs (*n* = 4 or 5). **E** Schematic representation of the impact of IFNα-MSCs on abscopal B16F0 tumors. Mice received co-injection of MC38 cells (1.0 × 10^6^) and IFNα-MSCs (1.0 × 10^6^) in the left outer thigh, and B16F0 cells (1.0 × 10^6^) only in the right outer thigh. Tumor sizes of both sides were measured. **F**, **G** Tumor volumes of ipsilateral MC38 tumors (**F**) and contralateral B16 tumors (**G**). **H** Tumor weights of ipsilateral MC38 tumors and contralateral B16 tumors (*n* = 5). **I** Schematic representation of the impact of IFNα-MSCs on abscopal MC38 tumor. B16F0 cells (1.0 × 10^5^) with IFNα-MSCs (1.0 × 10^6^) were co-injected intramuscularly into left outer thigh, then re-challenged with B16F0 cells (2.0 × 10^5^) in right outer thigh 2 weeks later. **J** Tumor volumes of ipsilateral B16 tumors. **K** Tumor volumes of contralateral MC38 tumors. **L** Weights of ipsilateral B16 tumors and contralateral MC38 tumors (*n* = 5). **M** Schematic representation of the duration of the anti-tumor effect of IFNα-MSCs. Mice were co-injected intramuscularly with B16F0 cells (1.0 × 10^5^) and IFNα-MSCs (1.0 × 10^6^) in the left outer thigh. Two weeks later, mice were injected with B16F0 cells (2.0 × 10^5^) in the right outer thigh. **N**, **O** Volumes (**N**) and weights (**O**) of B16 tumors in the right outer thigh (*n* = 5). Data are shown as means ± SEM. *p < 0.05 and **p < 0.01.

We then examined whether local administration of IFNα-MSCs could initiate systemic anti-tumor immunity. To this end, a mouse bilateral tumor model in which B16F0 cells were inoculated to both outer thighs was employed to evaluate the systemic tumoricidal effect of IFNα-MSCs (Fig. 3A). In this model, the left outer thigh of C57BL/6 mice was co-injected with B16F0 cells and IFNα-MSCs at a ratio of 1:1, while the right outer thigh only inoculated with B16F0 cells. Intriguingly, the introduction of IFNα-MSCs in the left outer thigh significantly inhibited tumor growth in the right outer thigh (Fig. 3B–D), suggesting an induction of a far-ranging anti-tumor immunity by IFNα-MSCs.

We next sought to determine whether the abscopal effect of the anti-tumor activity is tumor specific. B16F0 and antigenically distinct MC38 cells were separately injected in the outer thighs of mice (Fig. 3E). We found that co-administration of IFNα-MSCs with MC38 cells failed to inhibit the growth of B16F0 tumor (Fig. 3F–H). Likewise, co-injection of B16F0 cells with IFNα-MSCs was unable to suppress MC38 tumor growth (Fig. 3I–L). Importantly, B16F0 cells co-injected with IFNα-MSCs in one thigh blocked the growth of B16F0 tumor on the opposite thigh that was induced at 2 weeks after tumor and IFNα-MSCs injection (Fig. 3M). Compared with counterparts, the tumor sizes and volumes were much smaller in IFNα-MSC-administrated mice, suggesting a long-lasting anti-tumor effect (Fig. 3N, O). Taken together, the tumoricidal activity initiated by IFNα-MSCs can act remotely in a tumor specific and long-lasting manner.

### CD8^+^ T cells are essential in mediating the anti-tumor effect of IFNα-MSCs

**Fig. 4:**
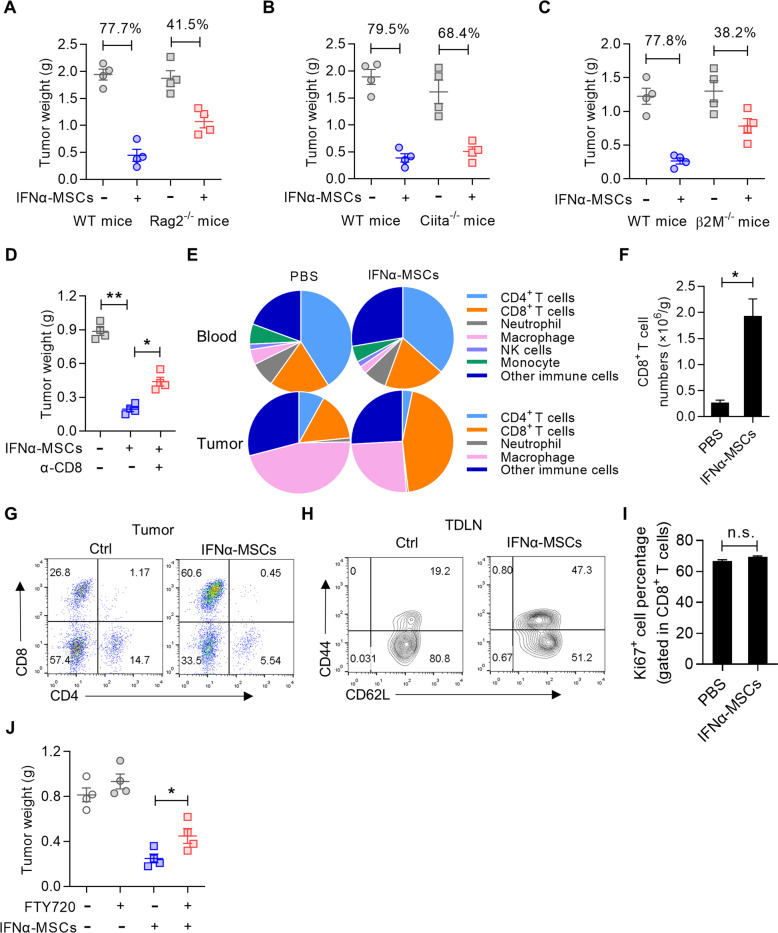
CD8^+^ T cells are essential for the anti-tumor effect of IFNα-MSCs. **A**–**C** The impact of IFNα-MSCs on tumor growth in immunodeficient mice. B16F0 cells were intramuscularly injected into wild type (WT), Rag2^−/−^ (**A**), Ciita^−/−^ (**B**), or β2m^−/−^ mice (**C**). On day 5, mice were *i.m*. injected with IFNα-MSCs (1.0 × 10^6^). On day 18, tumor weight was determined (*n* = 4). **D** The influence of depletion of CD8^+^ T cells on the anti-tumor effect of IFNα-MSCs. Mice were *i.m*. injected with B16F0 cells and administrated with IFNα-MSCs (1.0 × 10^6^), CD8 depleting Ab (α-CD8, clone number 2.43), or both. On day 18, tumor weight was detected (*n* = 4). **E** Profiling of immune cells in tumors and blood of tumor bearing mice with or without IFNα-MSC administration. On day 5, mice were *i.m*. injected with IFNα-MSCs (1.0 × 10^6^). On day 18, blood and tumor tissue were harvested for flow cytometric analysis. Shown are results of two independently experiments. **F** Numbers of tumor infiltrated CD8^+^ T cells in tumor bearing mice with or without IFNα-MSC administration. **G** Ratio of CD4^+^ and CD8^+^ T cells in tumors of mice with or without IFNα-MSC administration. **H** Expressions of CD44 and CD62L on CD8^+^ T cells in draining lymph node (TDLN) of tumor bearing mice with or without IFNα-MSC administration. **I** Expression of Ki67 in intra-tumoral CD8^+^ T cells of mice treated with or without IFNα-MSC administration. **J** The influence of blocking the egress of CD8^+^ T cells on the anti-tumor effect of IFNα-MSCs. Mice were intramuscularly injected with B16F0 cells and administrated with IFNα-MSCs (1.0 × 10^6^), FTY720 (1 mg/kg every 2 days) or both. On day 18, tumor weight was determined (*n* = 4). Data are shown as means ± SEM. n.s., no significance, *p < 0.05 and **p < 0.01.

Given the specific effects of IFNα-MSCs on limiting remote tumors and the induction of T cell accumulation at the tumor site (Supplementary Fig. S4A), we verified whether T cells are involved in the anti-tumor effects of these cells. The anti-tumor effects of IFNα-MSCs were evaluated in Rag2^−/−^ mice. We found that IFNα-MSCs exhibited partial impairment in tumor growth in Rag2^−/−^ mice (77.7% vs 41.5%) (Fig. 4A and Supplementary Fig. S4B). To examine which T cell population is critical, we further employed mice deficiency in MHC class II trans-activator (*Ciita*) [29] and β2-microglobulin (*β2m*) [30]. In Ciita^−/−^ mice, IFNα-MSCs exhibited similar ability in restraining tumor growth as WT mice (Fig. 4B), suggesting that CD4^+^ T cells are dispensable for the anti-tumor effect of IFNα-MSCs. However, IFNα-MSCs cannot efficiently suppress tumor growth in β2m^−/−^ mice (Fig. 4C), arguing the importance of CD8^+^ T cells. Antibody-mediated depletion of CD8^+^ T cells also attenuated the efficacy of IFNα-MSCs in suppressing tumor growth (Fig. 4D and Supplementary Fig. S4C). These data demonstrate that the anti-tumor effect of IFNα-MSCs mainly relies on CD8^+^ T cells.

Next, we profiled the immune cells in mice bearing B16F0 melanoma treated with or without IFNα-MSCs. The circulating and splenic T cells were not affected by IFNα-MSC administration (Fig. 4E and Supplementary Fig. S4D). However, the number and proportion of tumor infiltrated CD8^+^ T cells were dramatically increased in IFNα-MSCs treated mice (Fig. 4E–G). Moreover, CD8^+^ T cells in the tumor draining lymph node of mice treated with IFNα-MSCs exhibited a preference for the central memory phenotype (CD44^+^CD62L^+^) (Fig. 4H). By conducting Ki67 staining in intra-tumoral CD8^+^ T cells, we excluded the direct influence of IFNα-MSCs on T cell proliferation (Fig. 4I). Interestingly, when we utilized FTY720, a sphingosine 1-phosphate (S1P) receptor agonist, to block the egress of lymphocytes from lymphoid organs (Supplementary Fig. S4E), we found that the inhibition of IFNα-MSCs on tumor growth were dramatically impaired (Fig. 4J). Taken together, these results demonstrate that IFNα-MSCs facilitate the infiltration of CD8^+^ T cells in tumors, and thus lead to the suppression on tumor growth.

### IFNα-MSCs enhance CD8^+^ T cell infiltration into tumor via the CXCL10-CXCR3 axis

**Fig. 5:**
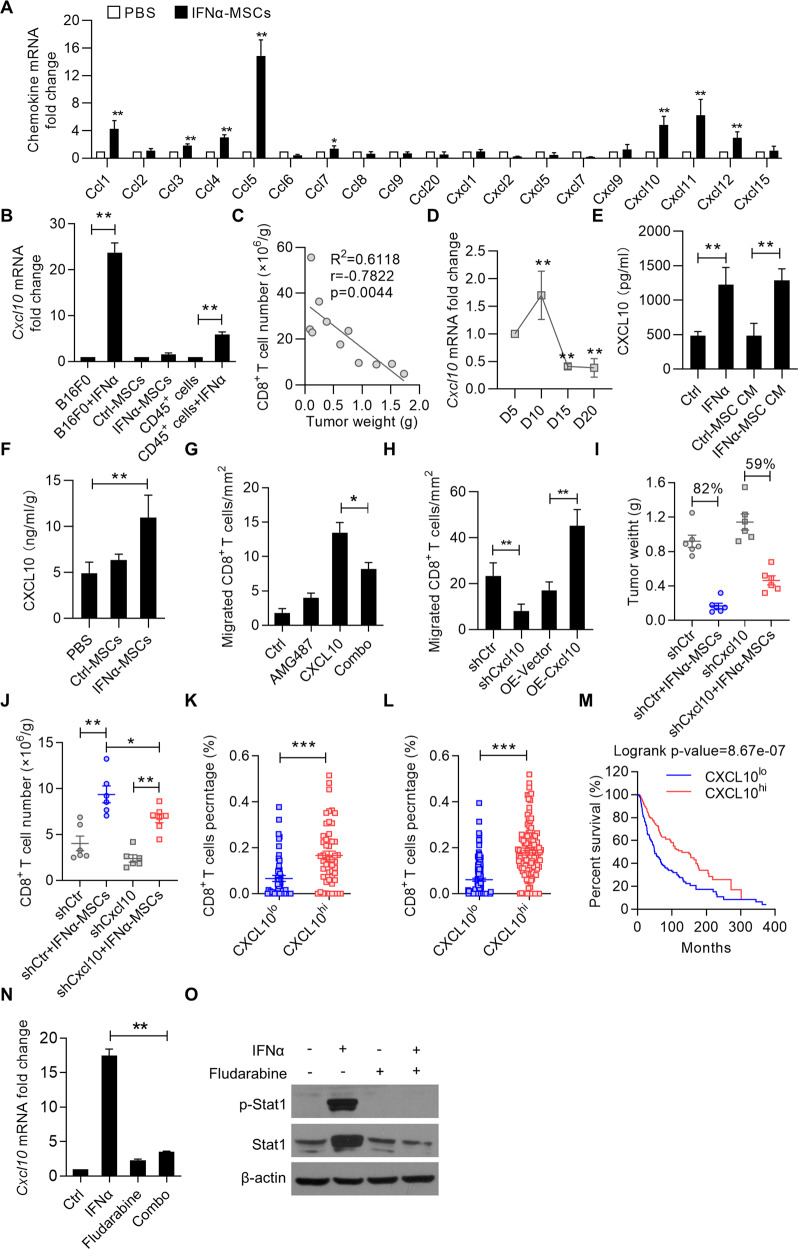
IFNα-MSCs enhance CD8^+^ T cell infiltration into tumors via the CXCL10-CXCR3 axis. **A** The chemokine expression profile in tumors of mice with or without IFNα-MSC treatment. **B** Expressions of *Cxcl10* in B16F0 cells, IFNα treated B16F0 cells, Ctrl-MSCs, IFNα-MSCs, CD45^+^ cells, and CD45^+^ cells treated with IFNα. **C** Scatterplots showing the correlation between the number of CD8^+^ T cells and tumor weight (*n* = 11). **D** The expression of *Cxcl10* in B16F0 tumors at indicated times. **E** Level of CXCL10 produced by B16F0 cells treated with IFNα, condition medium (CM) of Ctrl-MSCs or IFNα-MSCs. Level of CXCL10 was assessed by ELISA. **F** Level of CXCL10 in tumors of mice treated with Ctrl-MSCs or IFNα-MSCs. **G** Chemotaxis of CD8^+^ T cells in response to CXCL10. Naïve CD8^+^ T cells isolated from mouse spleen were stimulated by anti-CD3 and anti-CD28 for 48 h. Then, the migration of CD8^+^ T cells was assessed by the transwell system in the presence of CXCL10 (1 μg/ml), AMG487 (1 μM) or both for 4 h. **H** Chemotaxis of CD8^+^ T cells in response to the condition medium of B16F0 with *Cxcl10* knockdown or overexpression. B16F0 cells were transduced with lentivirus carrying *Cxcl10* shRNA or vector for gene encoding *Cxcl10*. Supernatant was obtained and used to test the impact on CD8^+^ T cell migration by transwell assay. OE, overexpression. **I** Tumor mass. Mice were inoculated with shCtr and shCxcl10 B16F0 cells and administered with IFNα-MSCs (1.0 × 10^6^ per mouse, *i.m*.) on day 5. **J** Numbers of CD8^+^ T cells in the tumor of mice inoculated with shCtr and shCxcl10 B16F0 cells and treated with IFNα-MSCs. **K**, **L** Comparison of the percentage of CD8^+^ T cells in melanoma between the *CXCL10* low (CXCL10^lo^) and *CXCL10* high (CXCL10^hi^) groups. Primary (**K**) and metastatic (**L**) melanoma patients based on CXCL10 expression were stratified into CXCL10^lo^ and CXCL10^hi^ cohorts (cutoff at 25%). The percentage of CD8^+^ T cells in melanoma were enumerated using CIBERSORT. **M** The overall survival analysis in *CXCL10*^*lo*^ and *CXCL10*^*hi*^ melanoma patients (cutoff at 25%). **N**, **O** Expression of *Cxcl10* (**N**) and Stat1 phosphorylation (**O**) in B16F0 cells treated with IFNα (1000 U/ml), Fludarabine (50 μM), or both. Data are shown as means ± SEM. *p < 0.05, **p < 0.01 and **p < 0.001.

To investigate the molecular mechanism(s) of IFNα-MSC-facilitated CD8^+^ T cell infiltration into tumors, we examined the chemokine expression profiles in matched tumor tissues. Compared to the control group, intra-tumoral injection of IFNα-MSCs upregulated the expressions of *Ccl3*, *Ccl4*, *Ccl5*, *Cxcl10*, *Cxcl11*, and *Cxcl12* in tumors (Fig. 5A). Among them, *Cxcl9*, *Cxcl10*, and *Cxcl11* are most related to the recruitment of CD8^+^ T cells, as CXCR3, the cognate receptor for CXCL9 and CXCL10, is highly expressed in tumor infiltrated CD8^+^ T cells (Supplementary Fig. S5A). To distinguish the main source of these chemokines, we detected the expression of chemokines in B16F0 cells, IFNα-treated B16F0 cells, Ctrl-MSCs, IFNα-MSCs, CD45^+^ cells, and CD45^+^ cells treated with IFNα by qPCR (Fig. 5B). A significantly higher levels of CXCR3 ligands, *Cxcl9* and Cxcl10, were observed in B16F0 cells stimulated with IFNα, but lower in Ctrl-MSCs or IFNα-MSCs (Supplementary Fig. S5B–D). Of note, only *Cxcl10* was highly expressed in B16F0 cells (Supplementary Fig. S5E). Given that an inversed relation between the tumor weight and CD8^+^ T cell infiltration and a dramatic downregulation of *Cxcl10* in melanoma at the late stage (Fig. 5C, D), we tested if addition of IFNα or IFNα-MSCs could enhance the expression of CXCL10 in B16F0 cells. Indeed, a significant enhancement of CXCL10 was observed in B16F0 cells treated with conditional medium of IFNα-MSCs, to a comparable level of that in IFNα treated B16F0 cells (Fig. 5E). Furthermore, IFNα-MSC administration elevated the concentration of CXCL10 in tumors (Fig. 5F).

By employing a transwell assay, we verified the role of CXCL10 in mediating CD8^+^ T cell migration via CXCR3 (Fig. 5G and Supplementary Fig. S5F). Additionally, we engineered B16F0 cells with *Cxcl10* knockdown (shCxcl10) or *Cxcl10* overexpression (OE-Cxcl10) using lentivirus transfection. We found that shCxcl10 B16F0 cell culture medium diminished, while OE-Cxcl10 B16F0 cell culture medium promoted CD8^+^ T cell migration (Fig. 5H). When compared with control B16F0 tumor, shCxcl10 B16F0 tumor showed compromised response to IFNα-MSC treatment in vivo (Fig. 5I). Consistently, knockdown of *Cxcl10* in B16F0 cells impaired IFNα-MSC-induced CD8^+^ T cell infiltration into tumor (Fig. 5J). We also found that primary melanoma or metastatic melanoma patients with higher levels of *CXCL10* expression exhibited more CD8^+^ T cell infiltration (Fig. 5K, L). In addition, high expression of *CXCL10* in patients suffering melanoma is an indicator of a good prognosis (Fig. 5M). These data strongly argue that melanoma cells can be influenced by IFNα released from IFNα-MSCs to produce CXCL10 to recruit CD8^+^ T cells into tumors.

We then investigated the molecular mechanisms that are involved in IFNα inducing CXCL10 expression. It has been reported that the NF-κB signaling pathway regulates CXCL10 expression [31]. However, using NF-κB inhibitor (PDTC) or IKK inhibitor (BAY 11-7082) to treat B16F0 cells showed little influence on IFNα-induced *Cxcl10* expression (Supplementary Fig. S5G). We next tested whether transducers and activators of transcription (Stats) in B16F0 cells mediate IFNα-induced CXCL10 expression. To achieve this, we used inhibitors (Fludarabine and Stattic) to block Stat1 and Stat3, respectively. We found that Stat1, but not Stat3, mediates CXCL10 expression induced by IFNα in B16F0 cells (Fig. 5N, O and Supplementary Fig. S5H). Together, IFNα-MSCs act on B16F0 cells and drive CD8^+^ T cell accumulation in the tumor through the IFNα-Stat1-CXCL10-CXCR3 axis.

### IFNα-MSCs potentiate the cytotoxicity of CD8^+^ T cells via the Stat3 signaling

**Fig. 6:**
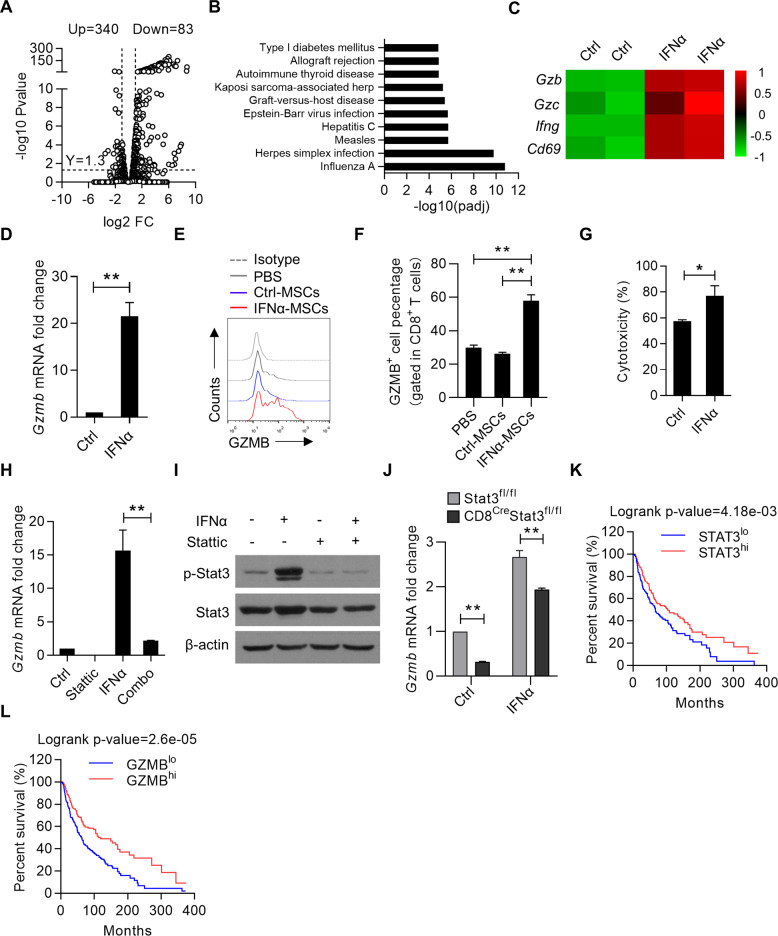
IFNα-MSCs potentiate CD8^+^ T cell cytotoxicity via the Stat3 signaling. **A** Volcano plot highlighting differentially expressed genes in IFNα treated CD8^+^ T cells compared with controls. CD8^+^ T cells were stimulated with PBS or IFNα (2 000 U/ml) for 48 h and subjected to RNA-seq analysis. **B** Gene ontology analysis of enriched transcripts in IFNα treated CD8^+^ T cells compared with controls. **C** Heatmap of the genes related to the activation and effector of CD8^+^ T cells. **D** The mRNA expression of *Gzmb* in CD8^+^ T cells treated with IFNα (2 000 U/ml) for 48 h. **E**, **F** Flow cytometric analysis of GZMB expression in tumor infiltrated CD8^+^ T cells. CD8^+^ T cells were isolated from tumor and stimulated by PMA and ionomycin in the presence of brefeldin A (BFA) for 4 h. Flow cytometry was used to analyze the expression of GZMB. **G** The cytotoxicity of intra-tumoral CD8^+^ T cells pretreated with IFNα. CD8^+^ T cells were isolated from B16F0 tumor and stimulated by anti-CD3 and anti-CD28 in the presence or absence of IFNα (2 000 U/ml) for 48 h. Then, CD8^+^ T cells were co-cultured with B16F0 cells at a ratio of 50:1 for 24 h. The cytotoxicity of CD8^+^ T cells was measured by lactate dehydrogenase (LDH) assay. **H** The regulation of STAT3 in IFNα induced *Gzmb* expression. CD8^+^ T cells were treated with IFNα (2 000 U/ml), Stattic (1 μM), an inhibitor of STAT3, or both for 12 h and determined the *Gzmb* expression by QPCR. **I** The effect of Stattic on STAT3 expression in CD8^+^ T cells. CD8^+^ T cells were treated with IFNα (2000 U/ml), Stattic (1 μM), an inhibitor of STAT3, or both for 0.5 h and examined the expression of Stat3 and phosphorylated Stat3 by immunoblotting. **J** The role of Stat3 in regulating GZMB expression in CD8^+^ T cells upon IFNα stimulation. CD8^+^ T cells were isolated from Stat3^fl/fl^ and CD8^Cre^Stat3^fl/fl^ mice and treated with PBS or IFNα (2000 U/ml) for 24 h. Cells were detected for *Gzmb* expression by QPCR. **K** The overall survival analysis in *STAT3*^*lo*^ and *STAT3*^*hi*^ melanoma patients in TCGA database. **L** The overall survival analysis in *GZMB*^*lo*^ and *GZMB*^*hi*^ melanoma patients. According to *STAT3* or *GZMB* expression, patients were stratified into two cohorts (cutoff at 25%). Data are shown as means ± SEM. *p < 0.05 and **p < 0.01.

Given the key role of CD8^+^ T cells in eradicating tumor cells, we sought to test the direct effect of IFNα on CD8^+^ T cells. Upon activation by anti-CD3 and anti-CD28, the addition of IFNα enhanced the expressions of CD25 and CD69 on CD8^+^ T cells, but no obvious influence on their proliferation (Supplementary Fig. S6A–C). To gain further insights into the mechanism(s) underlying how IFNα regulates CD8^+^ T cell function, we performed RNA-seq in CD8^+^ T cells with or without IFNα stimulation. IFNα imposed a distinct gene expression pattern on CD8^+^ T cells (Fig. 6A). Gene ontology analysis showed that the upregulated gene signature in IFNα treated CD8^+^ T cells was predominantly enriched for transcripts associated with “responses to virus”, “cellular responses to type I IFN” and “defense responses to virus” (Fig. 6B). Consistently, pathways related to “virus infection” and “immune response” were changed prominently upon IFNα treatment (Supplementary Fig. S6D). The heatmap data showed that IFNα treatment sharply increased the expression of Granzyme B (*Gzmb*), a gene signature for the cytotoxicity of CD8^+^ T cells (Fig. 6C). In line with this, IFNα or condition medium of IFNα-MSCs markedly promoted *Gzmb* expression (Fig. 6D and Supplementary Fig. S6E). Accordingly, administration of IFNα-MSCs to mice bearing B16F0 cells could dramatically promote the expression of GZMB in tumor infiltrated CD8^+^ T cells (Fig. 6E, F). In addition, these tumor infiltrated CD8^+^ T cells treated with IFNα exhibited stronger tumoricidal activity than those in mice of the control group (Fig. 6G).

We also investigated whether Stat(s) would be able to control the expression of GZMB in CD8^+^ T cells upon IFNα stimulation. Indeed, enhanced phosphorylation of Stat3 at Tyr705 was observed in IFNα treated CD8^+^ T cells, and this effect was markedly compromised by Stattic treatment (Fig. 6H, I). Both Stat1 inhibitor (Fludarabine) and Stat3 inhibitor (Stattic) were used to treat CD8^+^ T cells, in the presence or absence of IFNα. We found that IFNα-induced *Gzmb* expression is mediated by Stat3, but not Stat1 (Fig. 6H and Supplementary Fig. S6F). We further employed the Cre/Loxp system to specifically delete Stat3 in T cells in vivo. Compared to CD8^+^ T cells derived from Stat3^fl/fl^ mice, CD8^+^ T cells isolated from CD8^Cre^Stat3^fl/fl^ mice expressed lower level of *Gzmb*. Moreover, IFNα-induced *Gzmb* expression was impaired in CD8^+^ T cells with Stat3 deletion (Fig. 6J). By analysis of SKCM-TCGA data, we also found a positive correlation between *STAT3* and *GZMB* expression in human melanoma (Supplementary Fig. S6G). Consistently, melanoma patients with higher level expression of *STAT3* or *GZMB* suggested a good prognosis (Fig. 6K, L). Therefore, these data demonstrate that the release of IFNα by IFNα-MSCs reinforces the cytotoxicity of CD8^+^ T cells via regulation of the Stat3 signaling.

### IFNα-MSCs improve the efficacy of PD-L1 blockade

**Fig. 7:**
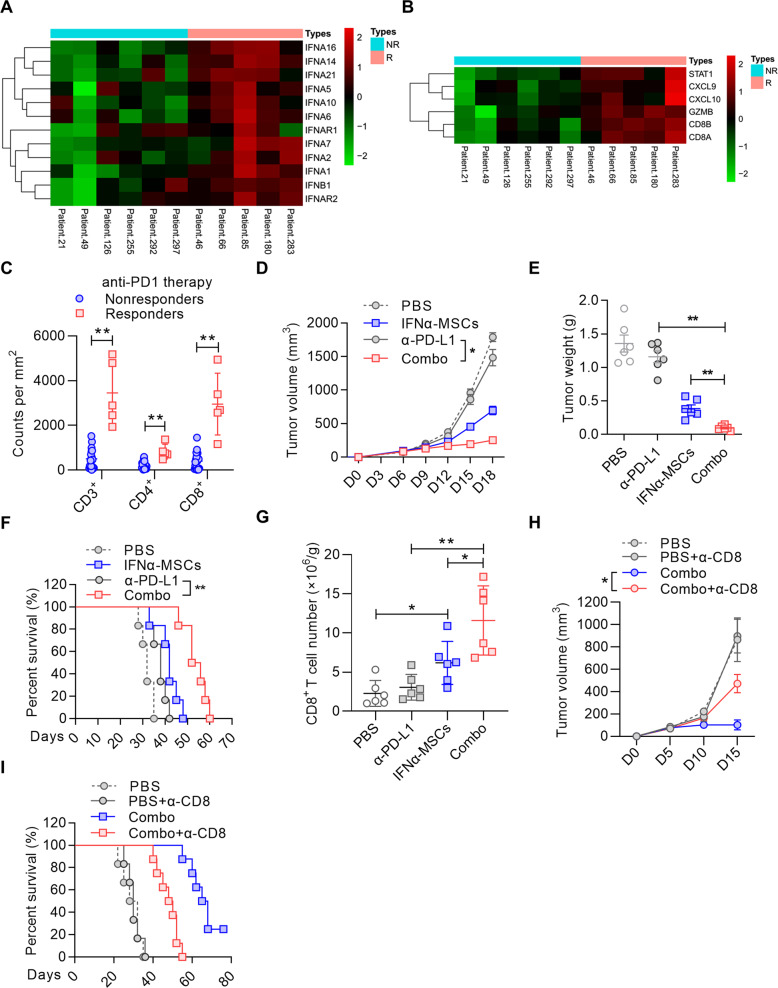
IFNα-MSCs synergize with PD-L1 blockade. **A** Comparison of *IFNA* and *IFNARs* expression in tumors from PD-1 blockade responders and non-responders. **B** Comparison of *STAT1*, *CXCL9*, *CXCL10*, *GZMB*, *CD8B*, and *CD8A* expression in tumors of PD-1 blockade responders and non-responders. **C** The density of CD3^+^, CD4^+^, and CD8^+^ T cells in the tumors of PD-1 blockade responders and non-responders. **D** The synergistic anti-tumor effect of IFNα-MSCs and PD-L1 blockade on tumor growth. B16F0 tumor bearing mice were administered with IFNα-MSCs (1.0 × 10^6^ per mouse, *i.m*.) or α-PD-L1 (100 μg per mouse, *i.p*.) or both (Combo) on day 5 after tumor initiation. Tumor volume was measured and calculated every 3 days (*n* = 4). **E** Mass of B16F0 tumors treated with IFNα-MSCs, α-PD-L1, or their combination (Combo) (*n* = 6). **F** The survival curves of B16F0 tumor bearing mice treated with IFNα-MSCs, α-PD-L1, or combo (*n* = 6). **G** Numbers of CD8^+^ T cells in the tumors of mice with treatment of IFNα-MSCs, α-PD-L1, or combo (*n* = 6). **H**, **I** Impairing the synergistic anti-tumor effects of IFNα-MSCs and PD-L1 blockade by CD8^+^ T cell depletion. Mice were inoculated with B16F0 cells and treated with IFNα-MSCs (1.0 × 10^6^) plus α-PD-L1 (100 μg per mouse), with or without administration of α-CD8 at 200 μg per mouse (*n* = 6 or 8). Tumor volume was measured and calculated every 5 days (**H**). The survival of B16F0 tumor bearing mice were monitored (**I**). Data are shown as means ± SEM. *p < 0.05, and **p < 0.01.

Lack of preexisting immune cell infiltration is an indicator of primary resistance to immune checkpoint blockade [32]. Given the powerful ability of IFNα-MSCs in deployment of CD8^+^ T cells into tumors, we hypothesized that combination of IFNα-MSCs and PD-L1 blockade may modulate the immune context of tumor and empower more potent anti-tumor effect. This notion is supported by the analysis of RNA sequencing data from PD-1 responders and non-responders, which showed that both *IFNA* and *IFNARs* were expressed at higher levels in patients responded well to the treatment of PD-1 blockade (Fig. 7A) [33]. Patients that respond to PD-1 blockade also exhibited increased expression of *STAT1*, *CXCL9*, *CXCL10*, *GZMB*, *CD8A,* and *CD8B*, which is consistent with our observation in tumor model with IFNα-MSC treatment (Fig. 7B). We re-analyzed their immunohistochemistry data and found that the density of CD3^+^, CD4^+^, and CD8^+^ T cells were prominently higher in anti-PD1 responders as compared to non-responders (Fig. 7C).

IFNα is one of the most potent inducers of PD-L1, which serves as a negative feedback mechanism to dampen immune responses [34]. Consistent with previous studies, we found that IFNα robustly induce PD-L1 expression in a dose-dependent manner in B16F0 cells (Supplementary Fig. S7A, B). In the mouse melanoma model, however, α-PD-L1 alone administration only had minor impact on the rate of tumor growth, suggesting simple blockade of the immune checkpoint is insufficient to elicit a powerful anti-tumor immune response. However, in comparison with the control group, mouse treated with the combination of IFNα-MSCs and α-PD-L1 profoundly decreased tumor growth (Fig. 7D, E) and increased survival time of tumor bearing mice over monotherapies (Fig. 7F). To better understand how the treatment with α-PD-L1 plus IFNα-MSC regulated the tumor immune response, we counted CD8^+^ T cells in matched tumors. Treatment with IFNα-MSCs and α-PD-L1 significantly increased CD8^+^ T cells in tumor (Fig. 7G). To further verify whether CD8^+^ T cells are responsible for the enhancement of anti-tumor efficacy of α-PD-L1 plus IFNα-MSCs, we depleted CD8^+^ T cells by intraperitoneal injection α-CD8 antibody (clone number 2.43). Depletion of CD8^+^ T cells diminished the anti-tumor effects of α-PD-L1 plus IFNα-MSCs, but had no effect in the untreated group (Fig. 7H). Moreover, the survival benefit induced by the combination treatment was comprised when CD8^+^ T cells were depleted (Fig. 7I). Collectively, these results indicate that administration IFNα-MSCs could strikingly enhance the responsiveness to PD-L1 blockade.

## Discussion

**Fig. 8:**
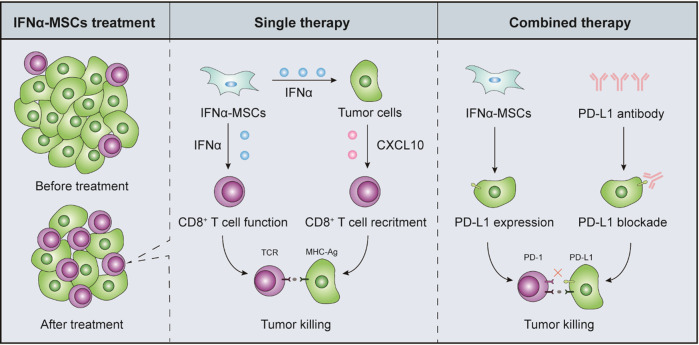
Schematic model depicting the mechanisms of anti-tumor effect by IFNα-MSCs. Administration of IFNα-MSCs stimulates tumor cells to produce CXCL10, which attracts the accumulation of CD8^+^ T cells in tumor sites. IFNα released by IFNα-MSCs also enhances GZMB expression in CD8^+^ T cells and potentiates their anti-tumor activities. More importantly, such remodeling on anti-tumor immune microenvironment by IFNα-MSCs can optimize the therapeutic effect of PD-L1 blockade.

The paucity of immune cells in tumors adversely correlates with patient prognosis. Remodeling tumor microenvironment holds great promise for cancer treatment. Here, we found that administration of IFNα-MSCs attracted CD8^+^ T cells into tumors and elicited anti-tumor activities. In the tumor microenvironment, IFNα-MSCs suppress tumor progression in an action of “killing two birds with one stone”: (i) Promoting CD8^+^ T cell infiltration into tumor tissue; (ii) Potentiating the cytotoxicity of CD8^+^ T cells in a tumor specific manner (Fig. 8). Of note, combination treatment of IFNα-MSCs and PD-L1 blockade synergistically suppresses tumor growth and significantly improves the survival of tumor bearing mice.

Consistent with previous studies, our analysis demonstrated that low levels of signals elicited by type I IFNs correlate with poor prognosis in melanoma patients. Therefore, reintroduction of type I IFNs or IFN signaling are principally important in combating tumor [35]. However, systemic administration of type I IFNs is always accompanied by severe adverse effects, limiting their clinical applications. In our previous studies, we equipped MSCs with IFNα and tested their ability to specifically deliver IFNα to solid tumors [27, 28]. Compared to IFNα, IFNα-MSCs were more effective in suppression of tumor growth. More importantly, IFNα-MSCs remodel anti-tumor immune microenvironment through a concerted action. In this scenario, IFNα-MSCs in tumor sites induce the production of CXCL10 in tumor cells which in turn mobilizes CD8^+^ T cell into tumors. Meanwhile, IFNα released by IFNα-MSCs enhances the expression of GZMB in CD8^+^ T cells and thus promotes their ability to eradicate tumor cells. Therefore, employment of IFNα-MSCs to modulate tumor microenvironment holds great promise in the treatment of solid tumors.

Clinically, primary tumors have more optional treatments, including surgery, chemotherapy, radiotherapy, and immunotherapy. However, the available treatments for unresectable or drug-resistant tumors are largely limited [36]. In fact, about >90% of mortality from cancer is attributable to subsequent metastases [37]. Thus, there is a desperate need to develop novel strategy to target local and distant tumors concurrently. In this study, by employing bilateral tumor model, we found that introduction of IFNα-MSCs in ipsilateral tumors could control the growth of contralateral tumors. Evidence that local administration of IFNα-MSCs contributed to a systemic anti-tumor effect was provided by the increase in CD44^+^CD62L^+^ CD8^+^ T cells in tumor draining lymph node observed in tumors injected with IFNα-MSCs. Therefore, administration of IFNα-MSCs may be an ideal therapeutic strategy to treat cancer patients with distal metastases.

Our study provides further insights into anti-tumor immunotherapies. A combination therapy of IFNα-MSCs and PD-L1 blockade demonstrated an enhancement in survival and tumor control. Such a combination reversed the paucity of immune cell infiltration in the tumor microenvironment, a major reason for the failure of immune checkpoint blockade treatment. Previous studies have demonstrated that promoting the infiltration of tumor antigen-specific CD8^+^ T cells in tumors can strengthen immunotherapy response [38, 39]. Activation of type I IFN signaling improved the efficacy of PD-1 blockade in melanoma featured by less immune cells [40]. Consistently, type I IFN fusion protein could optimize the therapeutic effects of PD-L1 blockade [14, 18, 41]. Together, these findings consolidate that targeted delivery of type I IFN into the tumor microenvironment is a feasible approach to enhance the therapeutic effect of immune checkpoint blockade treatment. Future investigations should be focused on exploring whether IFNα-MSC administration could optimize the efficacies of other anti-tumor strategies, such as tumor vaccination, radiotherapy, and chemotherapy [42–44].

IFNα treatment facilitates lymphocyte infiltration into tumors through augment of CXCL10 production by tumor cells. CXCR3, a receptor for CXCL9, CXCL10, and CXCL11, is expressed on T cells [45]. Activation of CXCR3 could induce T cell infiltration into inflamed sites [46]. However, CXCR3 is also expressed on melanoma cells. It has been reported that ligation with CXCL10 increased the metastasis of melanoma [47–49]. Therefore, strategies targeting CXCR3 to boost cytotoxic T lymphocyte recruitment should be carefully designed to avoid the hijack of melanoma cells.

It should be noted that type I IFNs have dramatic effects on the activation, migration, differentiation, function, and survival of innate immune cells, including dendritic cells, natural killer cells, macrophages, neutrophils, and monocytes [16, 50–55]. Type I IFNs stimulated antigen presenting cells to express high levels of MHC molecules and co-stimulatory molecules, such as CD80 and CD86 [56], which are critical in initiating and amplifying T cell activation. A recent publication also emphasized that delivery of type I interferon elicited an anti-tumor immunity via XCR1^+^ dendritic cells [51]. Therefore, the influence of IFNα produced by IFNα-MSCs on CD8^+^ T cells could involve its regulation on innate immune cells. Future investigation will examine the roles of dendritic cells and macrophages in the anti-tumor T cell immunity induced by IFNα-MSCs. In addition, MSCs are well-known in immunosuppression, raising the potential in orchestrating tumor immune microenvironment to facilitate tumor progression. In fact, the immunosuppression of MSCs should be evoked by IFNγ and TNF or IL-1 and can be totally abolished by the presence of IFNα [28]. We here demonstrate that MSCs equipped with IFNα can deploy tumoricidal CD8^+^ T cells in tumor microenvironment and synergistically enhance anti-tumor effect of α-PD-L1.

Together, our study highlights the potential to harness IFNα-MSCs to invigorate T cells and remodel tumor immune microenvironment, which should have beneficial effects in eradicating multiple types of tumors. Such approach provides insights into the application of immune checkpoint blockade, which need the preexisting T cell infiltration and/or presence of PD-L1 and PD-1. It is critical to examine if the strategies reported herein can be extended to other tumor types, especially those that are less immunogenic. Nevertheless, our study not only illustrates the molecular mechanism of the utilization of IFNα-MSCs in eradicating tumors, but also verifies an approach of using IFNα-MSCs to enhance the responsiveness to immune checkpoint blockade.

## Materials and methods

### Study design

The objective of this study was to investigate the therapeutic effect of IFNα-MSCs on tumor progression. To achieve this, we first used TCGA database to analyze the relationship between type I IFNs and their related signals and clinical outcome of patients with melanoma. Second, we constructed IFNα secreting MSCs and tested their anti-tumor effect in explanted and spontaneous tumors. By employment of specific gene knockout mice and antibody to delete certain type of immune cells, we confirmed that CD8^+^ T cells are indispensable for the therapeutic effects of IFNα-MSCs. We further verified the molecular mechanisms of IFNα in treatment of tumors. Last, we explored the synergistic effect of IFNα-MSCs and PD-L1 blockade on the suppression of tumor.

### Cells

MSCs were isolated from bone marrow of 6-week-old female C57/BL6 mouse tibia and femur according to the protocol previously described by our laboratory [57]. Ctrl-MSCs and IFNα-MSCs were constructed by lentivirus transfection as previously described [27]. MC38 cells were purchased from Kerafast (Boston, MA). B16F10 cells and MC38 cells were cultured in Dulbecco’s modified Eagle’s medium supplemented with 10% fetal bovine serum, 2 mM glutamine, 100 U/ml penicillin, and 100 μg/ml streptomycin. All cell lines were tested negative for Mycoplasma contamination and authenticated with short tandem repeat assays. Splenic or intra-tumoral CD8^+^ T cells were isolated using magnetic cell sorting kit (Miltenyi Biotec, Bergisch Gladbach, Germany). CD8^+^ T cells were cultured in RPMI-1640 medium supplemented with 10% fetal bovine serum, 2 mM glutamine, 100 U/ml penicillin, 100 μg/ml streptomycin, 1 mM sodium pyruvate, and 55 μM 2-mercaptoethanol (Gibco, Grand Island, NY).

### Mice

C57BL/6 mice were purchased from Shanghai Laboratory Animal Center of the Chinese Academy of Science (Shanghai, China). β2m^−/−^, Ciita^−/−^, Stat3^fl/fl^, and CD8-Cre mice were purchased from Jackson Laboratory. Rag2^−/−^ mice were purchased from Biomodel (Shanghai, China). MMTV-PyMT mice were kindly provided by Dr. Xiaoren Zhang of Shanghai Institute of Nutrition and Health of the Chinese Academy of Sciences. CD8^Cre^Stat3^fl/fl^ mice were obtained by crossing Stat3^fl/fl^ mice with CD8-Cre mice. All mice were maintained under specific pathogen-free condition and were performed in compliance with NIH Guide for the Care and Use of Laboratory Animals (National Academies Press, 2011) and the ARRIVE guidelines. Furthermore, all experiments were approved by the Institutional Animal Care and Use Committee of the Institute of Nutrition and Health, Shanghai Institutes for Biological Sciences of Chinese Academy of Sciences. A total of 8-week-old mouse was randomly divided into different groups (6–8 mice/group). The animals successfully inoculated with tumor were included in this study. The animals were excluded if the mice rejected tumors or if the animal died prematurely. Considering sex as a biological variable in research, animals were matched for gender in each experiment. For each animal study, due to powerful anti-tumor activity of IFNα-MSCs, the experimenter could not be blinded to whether the animal was injected with Ctrl-MSCs or IFNα-MSCs. 724 mice were used in this study: C57BL/6 mice (*n* = 600), MMTV-PyMT mice (*n* = 50), Rag2^−/−^ mice (*n* = 10), Ciita^−/−^ mice (*n* = 12), β2m^−/−^ mice (*n* = 12), Stat3^fl/fl^ mice (*n* = 20), and CD8^Cre^Stat3^fl/fl^ mice (*n* = 20).

### Reagents

Specific antibodies used for flow cytometry: CD3 (11-0031-85), CD45 (11-0451-85), CD4 (45-0042-82), CD8 (17-0081-83), CD11b (17-0112-83), Ly6C (17-5932-82), CD49b (12-5971-82), NK1.1 (17-5941-82), CD69 (17-0691-82), CD25 (17-0251-83), CXCR3 (17-1831-82), CXCR4 (12-9991-82), CD44 (12-0441-83), CD62L (17-0621-83), F4/80 (12-4801-82), CD11c(35-0114-82) were obtained from eBioscience Inc (La Jolla, CA). Antibodies to Ly6G (127616), Ki67 (652410), GZMB (515406), and CCR5 (107008) were purchased from BioLegend Inc (La Jolla, CA). Antibodies to Stat1 (9172 S), p-Stat1 (9171 L), p-Stat3 (9145 S), Stat3 (4904 S) and β-actin (4970 S) were purchased from Cell Signaling Technology (Danvers, MA). Small molecule inhibitors, BAY11-7082, PDTC, and AMG487 were purchased from MedChemExpress (Shanghai, China). Fludarabine, Stattic and FTY720 were obtained from TargetMol (Shanghai, China). Recombinant mouse IFNα were obtained from PBL Assay Science (New Brunswick, NJ). Recombinant mouse CXCL10 were obtained from Thermo Fisher Scientific (Waltham, MA). Mouse Cxcl10 shRNA lentiviral particles (shCxcl10: 5’- TTGATGGTCTTAGATTCCGGA-3’) and overexpressing particles (pSLenti-EGFP-P2APuro-CMV-MCS-3Flag and pSLenti-EGFP-P2APuro-CMV-Cxcl10-3Flag) were purchased from OBiO Technology (Shanghai, China).

### Tumor models

B16F0 and MC38 tumor models: B16F0 cells or MC38 cells (1 × 10^6^) were intramuscularly inoculated into outside thigh. Ctrl-MSCs or IFNα-MSCs (1 × 10^6^) were locally injected into peritumoral tissue every 3 days. Mice were inspected daily and euthanatized when tumor burden started to significantly affect their mobility. B16F10 mouse melanoma model: B16F10 cells (5 × 10^5^) were intravenously injected into C57BL/6 mice. On day 7, they also received intravenous injection of IFNα-MSCs (5 × 10^5^). Spontaneous mammary cancer model: MMTV-PyMT mice at 4 weeks old were treated with PBS, Ctrl-MSCs (3 × 10^5^) or IFNα-MSCs (3 × 10^5^) twice a week. The survival times of tumor bearing mice were recorded and plotted. Mouse melanoma with α-PD-L1 treatment: B16F0 cells (1 × 10^6^) were intramuscularly inoculated into the outside thigh. On day 5, these mice also received PD-L1 antibody (100 μg, clone 10F.9G2) and IFNα-MSCs (1 × 10^6^). Tumor volumes (volume = 0.5 × length × width^2^) were measured every 2 or 3 days.

### Isolation and activation of splenic CD8^+^ T cells

Naive CD8^+^ T cells were isolated from the mouse spleen using immunomagnetic separation beads (Miltenyi Biotec, Bergisch Gladbach, Germany). These CD8^+^ T cells were then seeded into 96-well plates (3 × 10^5^/well) pre-coated with 2.5 μg/mL anti-CD3 (16-0032-86, eBioscience) with the addition of soluble 1 μg/mL anti-CD28 (16-0281-86, eBioscience). The CD8^+^ T cells were activated for 2 or 3 days and used for the respective experiments.

### CFSE staining

Naïve CD8^+^ T cells were isolated from mouse spleen and were stained with 5 μM CFSE (Invitrogen, Carlsbad, CA) for 10 min. CFSE-labeled CD8^+^ T cells were stimulated with anti-CD3 (2.5 μg/mL)) and anti-CD28 (1 μg/mL) in the presence or absence of IFNα for 72 h. Flow cytometry was used to analyze CFSE intensity reduction as an indicator of cell proliferation.

### Stimulation of intra-tumoral CD8^+^ T cells in vitro

B16F0 tumor was homogenized by pressing through 70 μm cell strainers. After washing and centrifugation, cells were resuspended in 35% Percoll solution and layered on 70% Percoll followed by centrifugation at 2000 rpm for 20 min at room temperature. Lymphocytes were collected from the interface and washed with RPMI-1640 medium. Then, the CD8 MicroBeads kit was used to isolate CD8^+^ T cells according to the manufacturer’s instruction. Isolated CD8^+^ T cells were seeded in 96-well plates in the presence of α-CD3/CD28 antibodies or PMA (50 ng/ml) plus ionomycin (1 μg/ml) and incubated with Brefeldin A (BFA) for 4 h before stained for surface markers and intracellular cytokines.

### Cytokine measurement

The levels of IFNα and CXCL10 in serum or culture medium were determined by kits from eBioscience according to manufacturer’s indications.

### Western blotting analysis

Total protein was extracted from cells with RIPA lysis buffer (Beyotime, Shanghai, China). The protein concentration of each sample was determined by BCA Protein Assay (Thermo Scientific). Twenty μg proteins were loaded and separated on SDS-PAGE. After transferred onto PVDF membrane and blocked with 5% defatted milk powder, specific primary antibodies against p-Stat1, Stat1, p-Stat3, Stat3, and β-actin were used for specific protein detection and HRP-conjugated secondary antibodies were used to reveal specific bindings. The staining was detected with the ECL system (Millipore).

### Real-time PCR

Total RNA was extracted from cells and animal tissues using the Trizol kit (Invitrogen) or RNA easy mini kit (QIAGEN, Hilden, Germany) according to the manufacturer’s protocols. qPCR RT master mix kit (Takara, Kyoto, Japan) was used to synthesize cDNA. The qPCR analysis was performed using a FastStart Universal SYBR Green Master (Roche). Sequences of primers used are listed in Supplementary Table 1.

### Hematoxilin & eosin and immunofluorescence staining

Tissues from tumor bearing and tumor-free mice were collected and fixed in 4% paraformaldehyde overnight. The samples were sequentially dehydrated with ethanol. After treatment with xylene for 20 min twice, samples were embedded in paraffin. Then, the samples were sectioned at 5 μm thickness and stained with hematoxylin and eosin. For CD3 staining, the sections were incubated with rabbit anti-CD3 after deparaffinization, followed by incubation with Alexa Fluor 488 conjugated goat anti-rabbit IgG in dark. After DAPI staining, images were taken using a Zeiss Observer Z1 (Carl Zeiss).

### Cell cycle and apoptosis analysis

Annexin V/propidium iodide staining (eBioscience) was performed to assess apoptotic and necrotic cells. Briefly, B16F0 cells were treated with IFNα, Ctrl-MSC CM, or IFNα-MSC CM for 2 days. Annexin V/propidium iodide staining were carried out according to the manufacturer’s instructions. Cells were collected and washed with PBS, then analyzed using a BD FACS Caliber flow cytometer (BD Biosciences). The data were analyzed by using FlowJo software.

### RNA sequencing and analysis

Naïve splenic CD8^+^ T cells were isolated by immunomagnetic separation beads (Miltenyi Biotec, Bergisch Gladbach, Germany), and stimulated with anti-CD3 and anti-CD28 in the presence or absence of IFNα (2000 U/ml) for 48 h. Total RNA was isolated using Trizol reagent (Ambion). NEBNext^®^ UltraTM RNA Library Prep Kit for Illumina^®^ (NEB, USA) was used for generating sequencing libraries, and samples were sequenced on the Illumina HiSeq platform. Sequencing reads were mapped to the mouse reference genome using Hisat2 v2.0.5. edgeR was used for the normalization and identification of differentially expressed genes. The resulting *P*-values were adjusted using the Benjamini and Hochberg’s approach for controlling the false discovery rate. Genes with an adjusted *P*-value < 0.05 and │log2 (fold change) │> 1 were considered as differentially expressed.

### Data mining

TCGA-SKCM data was downloaded from the internet (http://gdac.broadinstitute.org/). All types of *IFNA* and *IFNARs* were selected for expression analysis. For CD8^+^ T cell infiltration analysis, *IFNAR1*, *IFNAR2*, and *CXCL10* expressions were cut off at 25%. The CIBERSORT analytical tool was used to enumerate CD8^+^ T cell number in melanoma tissues (https://cibersortx.stanford.edu/). For survival analysis, *IFNAR1*, *IFNAR2*, *JAK1*, *TYK2*, *STAT1*, *STAT2*, *IRF9*, *MIX1*, *CD8A*, *CXCL10*, *STAT3*, and *GZMB* expressions were cut off at 25% and plotted by using OncoLnc (http://www.oncolnc.org/). We used the existing data sets to investigate the link between *IFNA* and *IFNAR* expressions to PD-1 antibody treatment and generated Fig. 7A, B, C [33].

### Statistical analysis

Data are presented as means ± SEM as specified in the figure legends and analyzed with GraphPad Prism 8. The number of mice used per treatment group is indicated as “*n*” in the corresponding figure legends. Student’s *t*-test (two tailed) and Log-rank (Mantel–Cox) test were used for statistical analysis. Significant differences are indicated as follows: n.s., no significance, *p < 0.05, **p < 0.01, and ***p < 0.001.

## Supplementary information

Supplementary Table 1

Supplementary data

## Data Availability

No statistical methods were used to predetermine sample size. We thus estimated the sample size empirically. No random methods were used in this study. Investigators were not blinded to allocation during experiments. The authors declare that all relevant data of this study are available within the article or from the corresponding author on reasonable request. The RNA-seq data are openly available in Gene Expression Omnibus (GEO), GSE184918.
