# Supplementary material for: Mesenchymal stromal cells equipped by IFNα empower T cells with potent anti-tumor immunity
Source: Oncogene. 2022 Feb 10;41(13):1866–81. doi: 10.1038/s41388-022-02201-4 (PMC8956510; doi:10.1038/s41388-022-02201-4)
Supplement: Supplementary file 2 — Supplementary data [file 41388_2022_2201_MOESM2_ESM.docx]

**Supplementary information**

**Mesenchymal Stromal Cells Equipped by IFNα Empower T Cells**

**with Potent Anti-tumor Immunity**

Tao Zhang^1^, Yu Wang^1^, Qing Li^1^, Liangyu Lin^1^, Chunliang Xu^1^, Yueqing Xue^1^, Mingyuan Hu^1^, Yufang Shi^1, 2,*^ and Ying Wang^1,*^

**Supplementary Figure Legends:**

**Fig. S1** **Analysis of *IFNAR* expression in melanoma patients.** **A** Analysis of *IFNAR1* expression in melanoma patients. **B** Analysis of *IFNAR2* expression in melanoma patients. **C** Comparison of *IFNAR1* expression in normal skin, nevus and melanoma using the GSE3189 dataset. **D** The percentages of CD8^+^ T cells in metastatic melanoma of patients with low *IFNAR1* or high *IFNAR1* expression. *IFNAR1* expression was cutoff at 25%, and CIBERSORT was used to analyze the percentage of CD8^+^ T cells in metastatic melanoma based on TCGA-SKCM dataset. ***p* < 0.01.

**
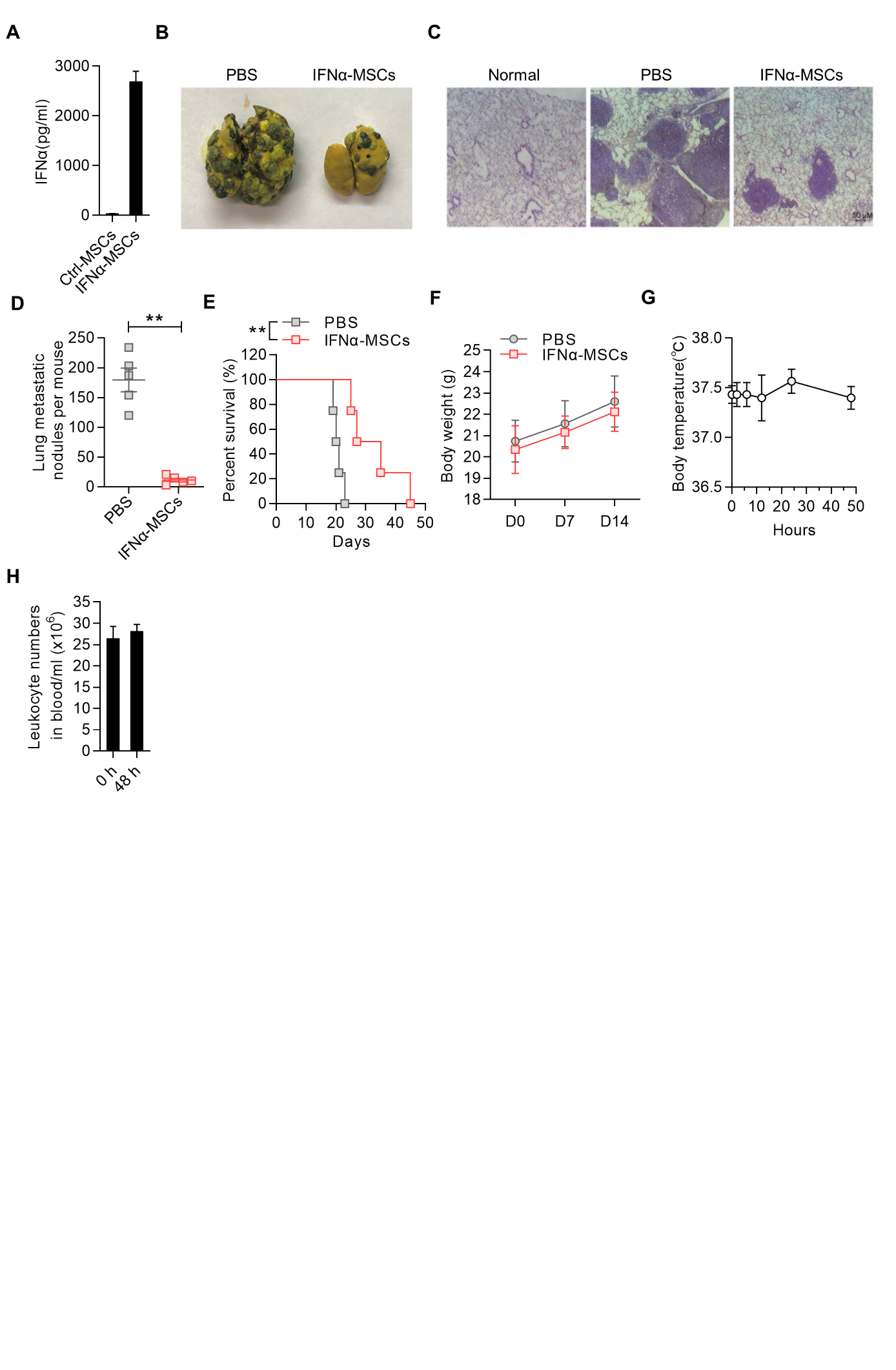
**

**Fig. S2** **Assessment of the toxicity of IFNα-MSCs.** **A** IFNα concentration in the culture medium of Ctrl-MSCs and IFNα-MSCs detected by ELISA assay. **B** Representative images of lungs treated with PBS or IFNα-MSCs. B16F10 cells (5 × 10^5^) were intravenously injected into mice. On day 7, mice received IFNα-MSCs intravenously (5 × 10^5^). At indicated timepoints, the lung tissues were harvested and the survival time was recorded. **C** Hematoxilin & eosin staining of the lung sections on day 21. **D** Quantification of the metastatic nodules in the lungs of mice treated with PBS or IFNα-MSCs (*n* = 5). **E** The survival curves of mice treated with PBS or IFNα-MSCs (*n* = 4). **F** Changes of body weight of mice treated with or without IFNα-MSCs. **G** Changes of body temperature of mice treated with IFNα-MSCs. **H** Numbers of leukocytes in peripheral blood of mice with IFNα-MSC administration. Data are shown as means ± SEM. ***p* < 0.01.

**
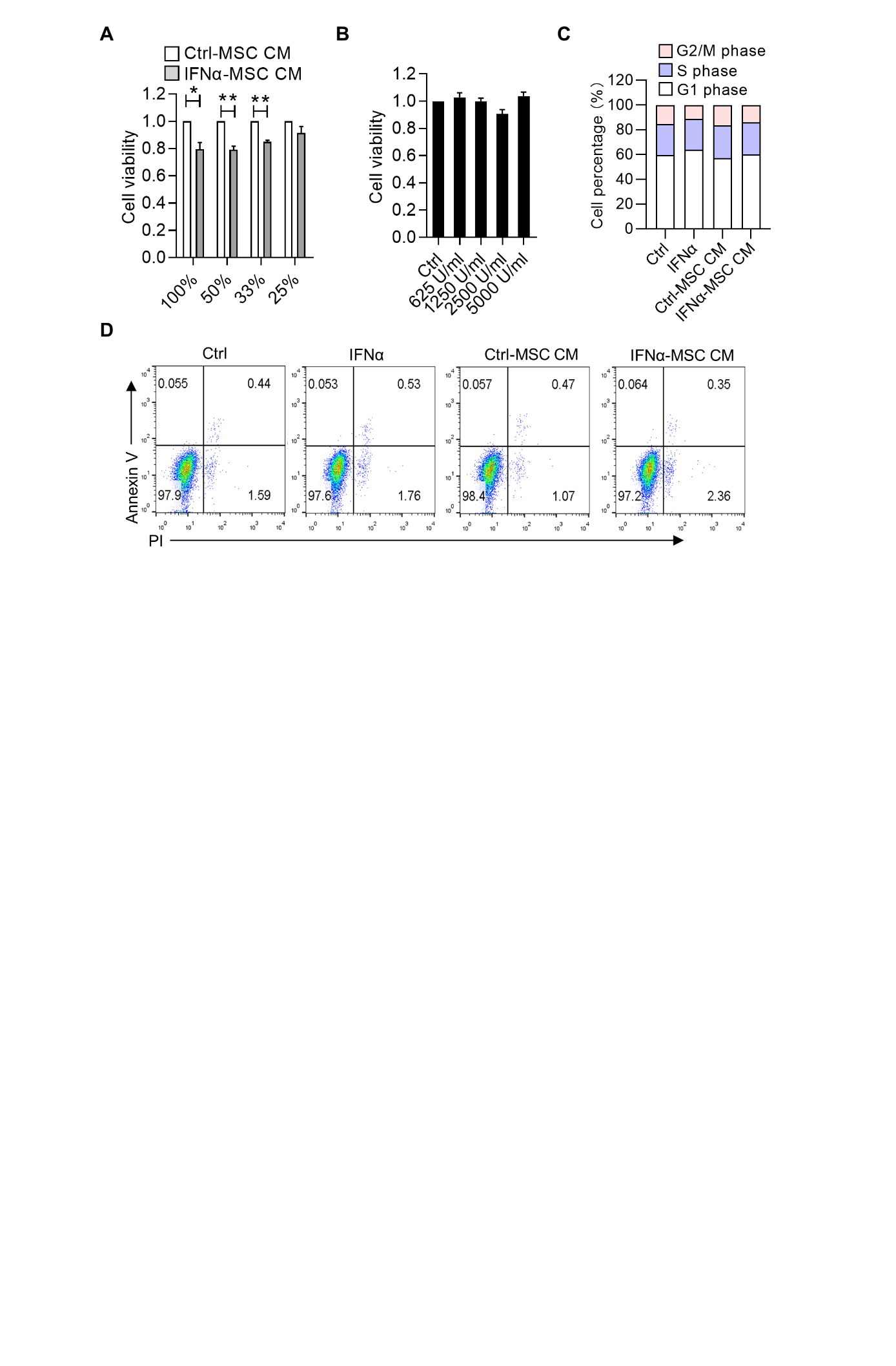
**

**Fig. S3 The anti-tumor effect of IFNα-MSCs is independent on the direct killing effect of IFNα on tumor cells.** **A** Cell viability. B16F0 cells were treated with the conditioned medium of Ctrl-MSCs (Ctrl-MSC CM) or IFNα-MSCs (IFNα-MSC) for 48 hours. **B** Cell viability. B16F0 cells were treated with IFNα at indicated concentration for 48 hours. Cell viability was assessed by MTS assay. **C** Analysis of cell cycle distribution of B16F0 cells treated with IFNα (1 000 U/ml), Ctrl-MSC CM or IFNα-MSC CM for 48 hours. **D** Analysis of apoptosis of B16F0 cells treated with IFNα (1 000 U/ml), Ctrl-MSC CM or IFNα-MSC CM for 48 hours. Data are shown as means ± SEM. **p* < 0.05 and ***p* < 0.01.

**
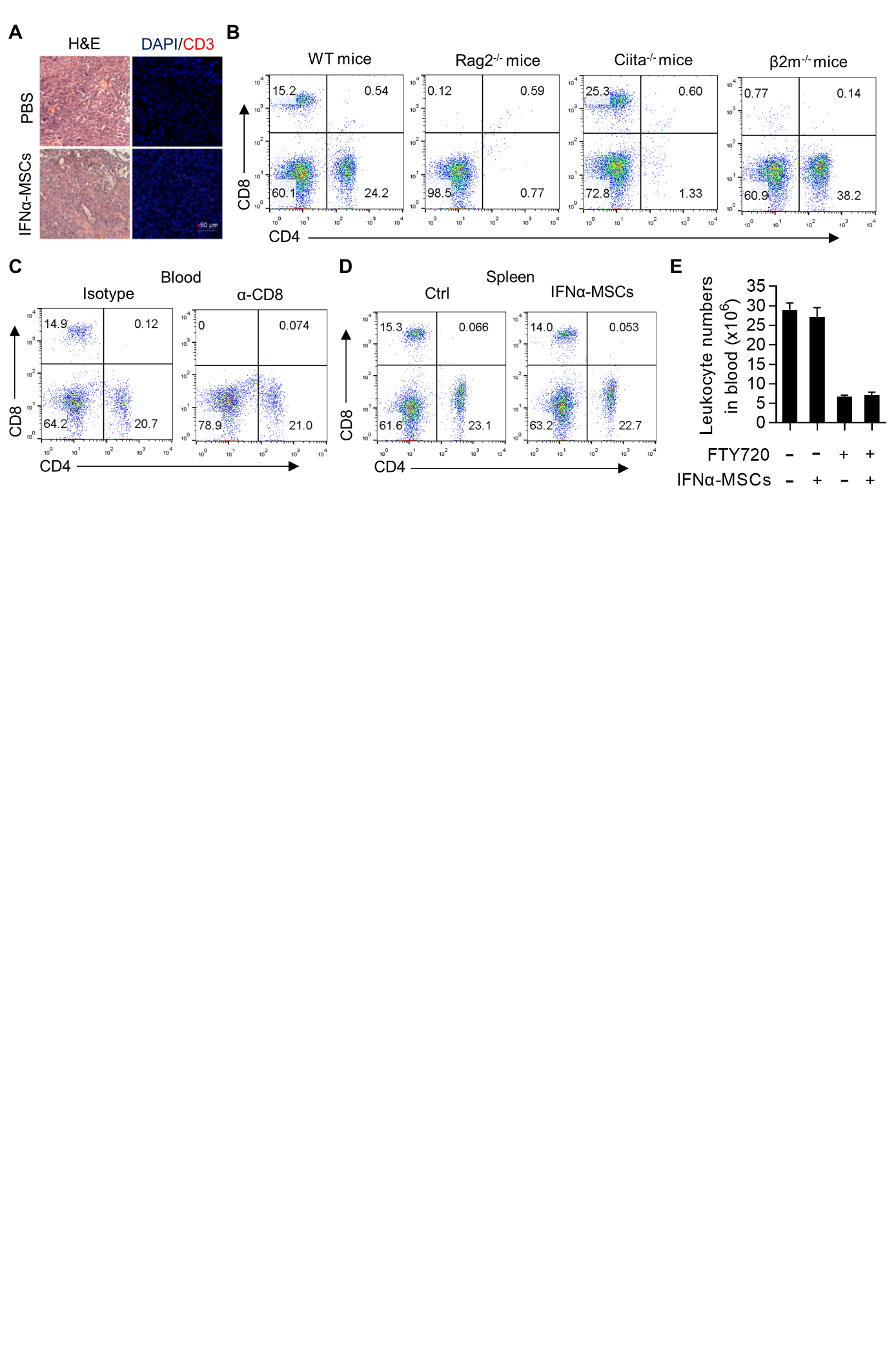
**

**Fig. S4 CD8^+^ T cells are important in mediating the IFNα-MSC-mediated tumoricidal activity.** **A** Immunofluorescent staining for CD3 positive cells in tumors treated with or without IFNα-MSCs. **B** The percentage of CD4^+^ and CD8^+^ T cells in wild type mice, Rag2^-/-^ mice, Ciita^-/-^ mice and β2m^-/-^ mice. **C** The percentage of CD4^+^ and CD8^+^ T cells in spleen after IFNα-MSC administration. **D** The leukocyte numbers in blood after FTY720 treatment. **E** The depletion efficiency of α-CD8 antibody. Data are shown as means ± SEM. **p* < 0.05 and ***p* < 0.01.

**
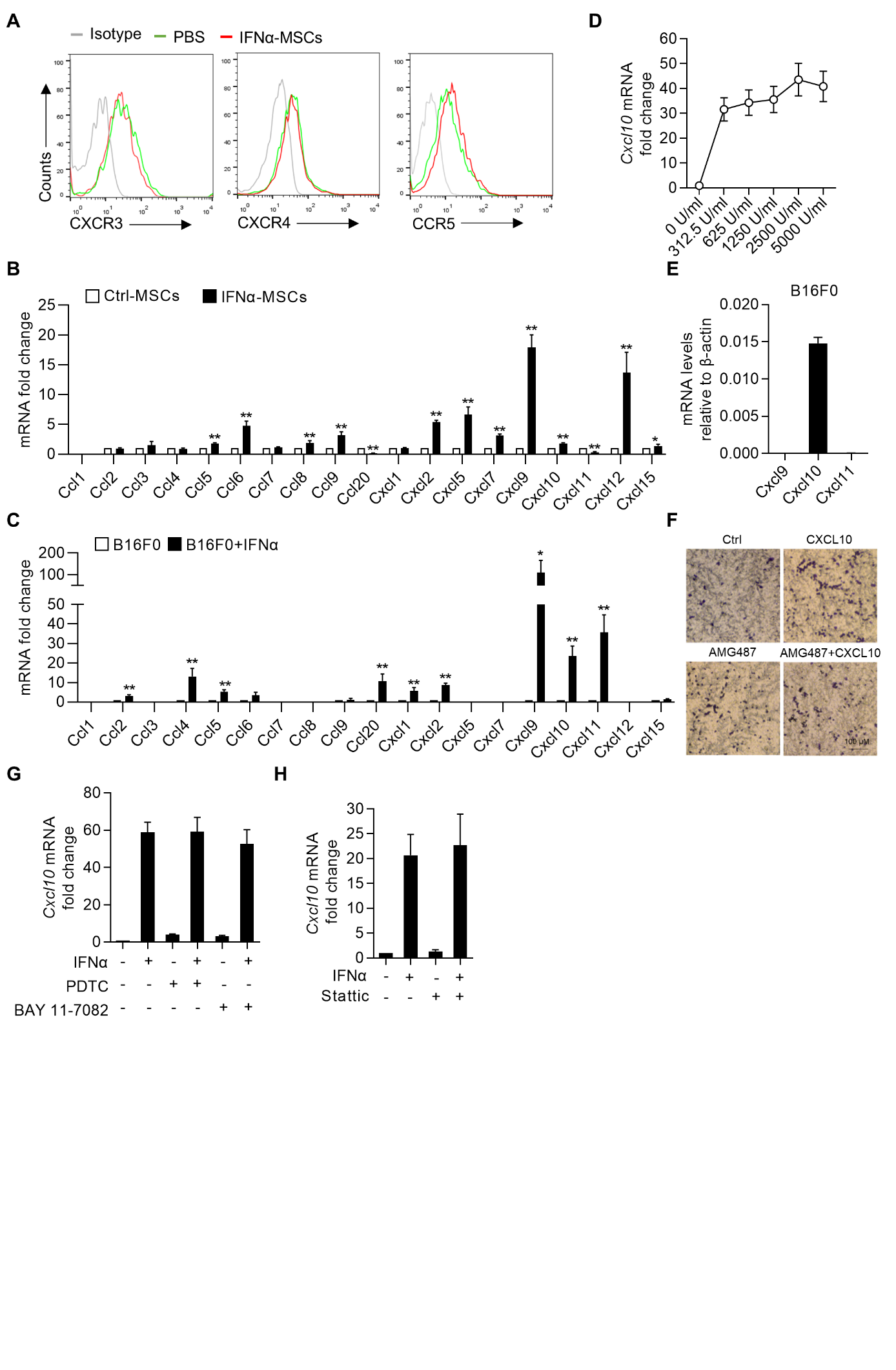
**

**Fig. S5 Enhanced CXCL10 expression in B16F0 cells treated with IFNα-MSCs recruits the infiltration of CD8^+^ T cells. A** The expression of CXCR3, CXCR4 and CCR5 on intra-tumoral CD8^+^ T cells was analyzed by flow cytometry. **B** Chemokine expression in B16F0 cells treated with or without IFNα (1 000 U/ml) for 12 hours. **C** Chemokine expression in Ctrl-MSCs and IFNα-MSCs. **D** The mRNA expression of *Cxcl10* in B16F0 cells treated with IFNα at indicated concentrations for 12 hours. **E** The expression of CXCR3 ligands in B16F0 cells. **F** Images of chemotaxis of CD8^+^ T cells upon CXCL10 (1 μg/ml) stimulation, with or without addition of AMG487 (1 μM). **G** The *Cxcl10* mRNA expression in B16F0 cells treated with IFNα (1 000 U/ml) for 12 hours, in presence of PDTC (10 μM) or BAY 11-7082 (5 μM). **H** The *Cxcl10* mRNA expression in B16F0 cells treated with IFNα (1 000 U/ml), Stattic (1 μM), or both for 12 hours. Data are shown as means ± SEM. **p* < 0.05 and ***p* < 0.01.

**
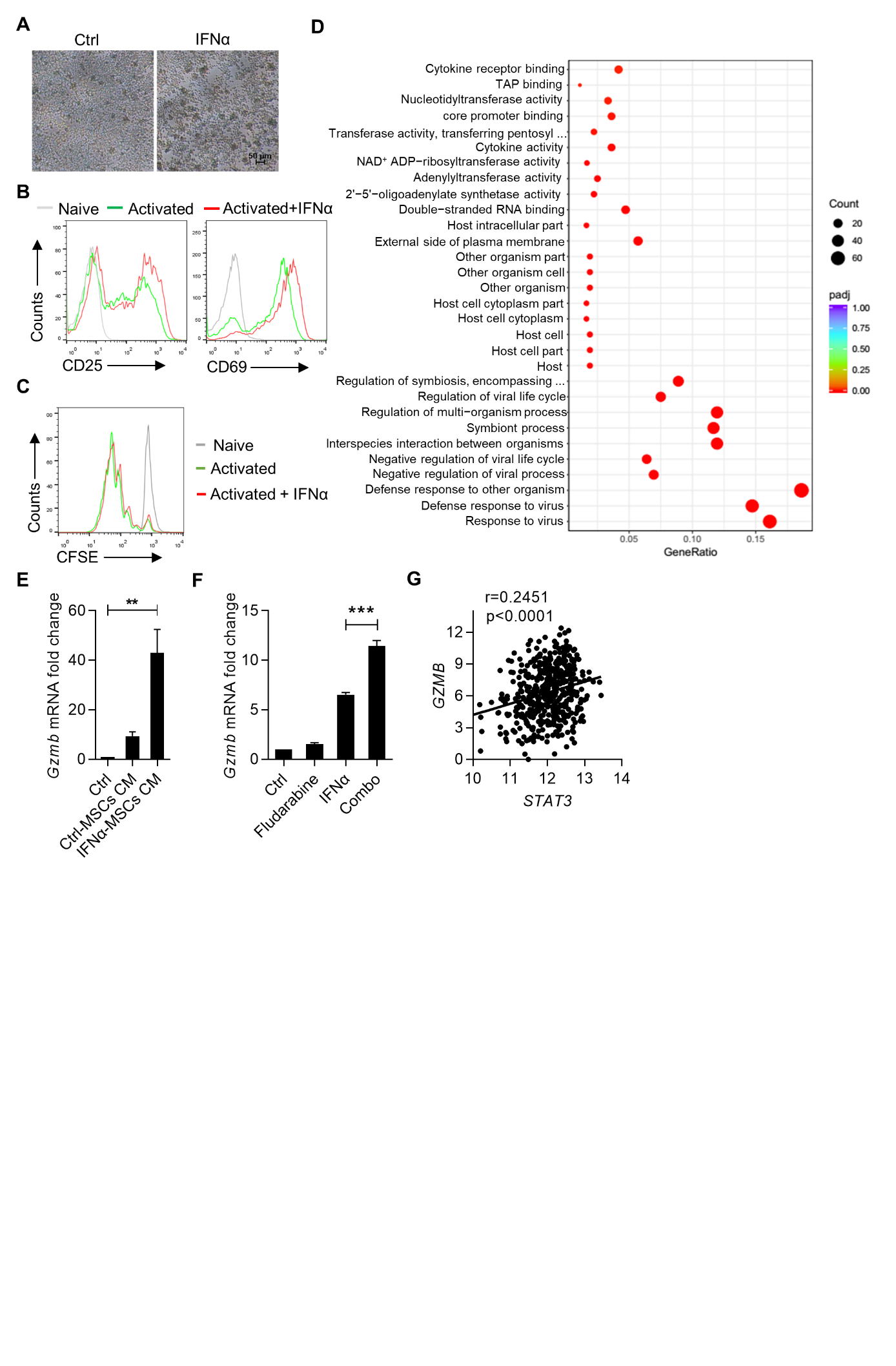
**

**Fig. S6** **IFNα-MSCs enhance the expression of GZMB in CD8^+^ T cells and their anti-tumor effect.** **A** Images of CD8^+^ T cells upon IFNα stimulation for 48 hours. Scale bar: 50 μM. **B-C** The activation and proliferation of CD8^+^ T cells upon IFNα stimulation. CD8^+^ T cells were stimulated with anti-CD3 and anti-CD28 antibodies, in presence or absence of IFNα for 72 hours. Cells were detected for the expression of CD25 and CD69 by flow cytometry (**B**). CD8^+^ T cell proliferation was assayed by CFSE staining (**C**). **D** Enriched signaling pathways in IFNα treated CD8^+^ T cells. **E** The *Gzmb* mRNA expression in CD8^+^ T cells cocultured with the condition medium of Ctrl-MSCs or IFNα-MSCs. **F** The influence of Stat1 on IFNα-induced *Gzmb* expression in CD8^+^ T cells. CD8^+^ T cells were treated with IFNα (2 000 U/ml), Fludarabine (50 μM), or both for 24 hours. **G** Scatterplots showing the correlation between *STAT3* and *GZMB* expression in TCGA-SKCM dataset. Data are shown as means ± SEM. ***p* < 0.01 and ****p* < 0.001.

**
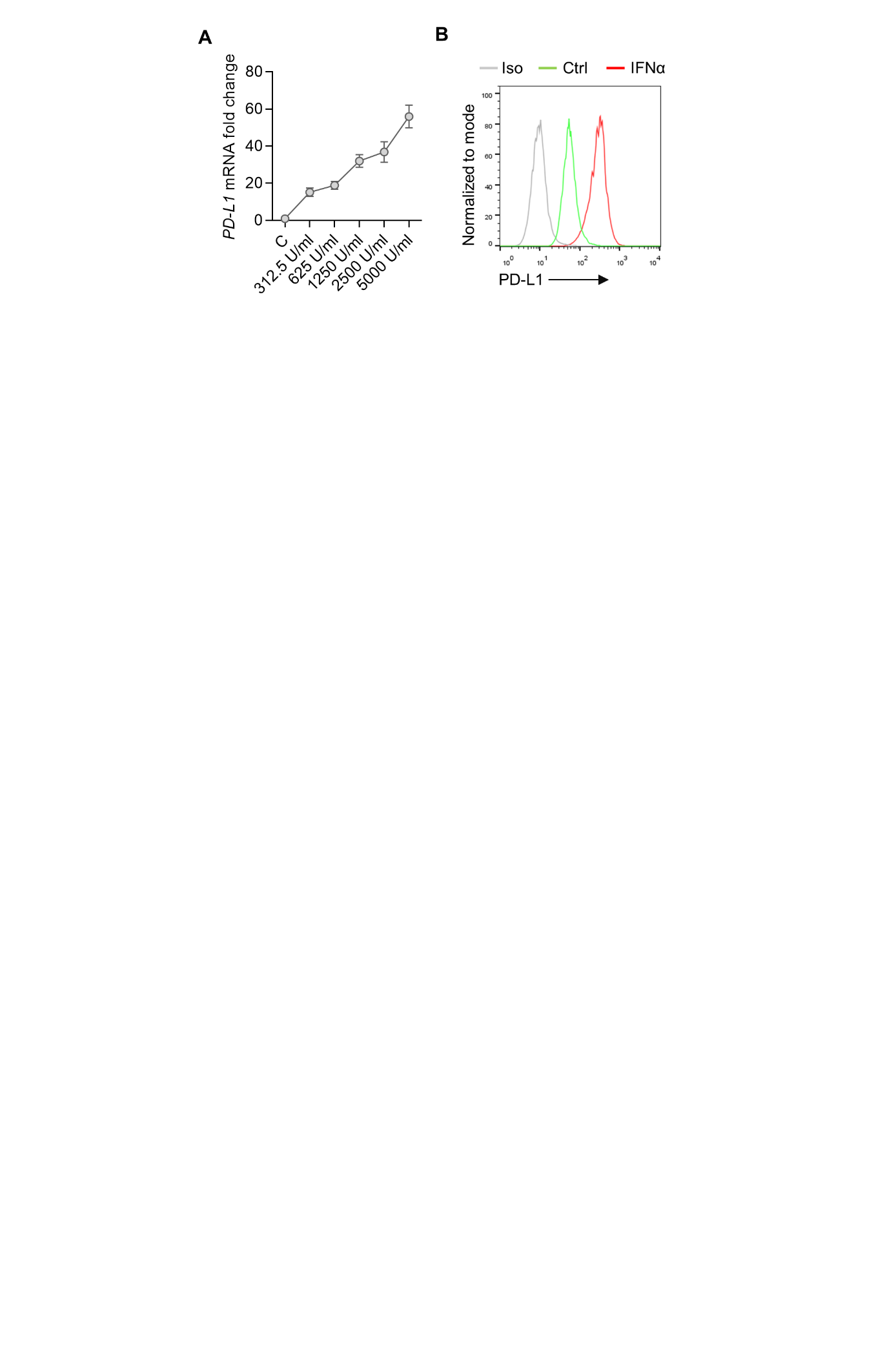
**

**Fig. S7** **IFNα induces PD-L1 expression.** **A** The *Pdl1* mRNA expression in B16F0 cells treated with different concentrations of IFNα for 24 hours. **B** Flow cytometric analysis of PD-L1 on B16F0 cells. B16F0 cells were incubated with IFNα (1 000 U/ml) for 48 hours. Data are shown as means ± SEM.
